# NMR and MS data for novel bioactive constituents from *Pugionium cornutum* L. Gaertn

**DOI:** 10.1016/j.dib.2020.105122

**Published:** 2020-01-11

**Authors:** Wenzhong Shi, Jingya Ruan, Yuanqiang Guo, Zhijuan Ding, Jiejing Yan, Lu Qu, Chang Zheng, Yi Zhang, Tao Wang

**Affiliations:** a Tianjin State Key Laboratory of Modern Chinese Medicine, 312 Anshanxi Road, Nankai District, Tianjin, 300193, China; b Tianjin Key Laboratory of TCM Chemistry and Analysis, Institute of Traditional Chinese Medicine, Tianjin University of Traditional Chinese Medicine, 312 Anshanxi Road, Nankai District, Tianjin, 300193, China; c Tianjin Key Laboratory of Molecular Drug Research, College of Chemistry, Nankai University, Tianjin, 300071, China

**Keywords:** Traditional Mongolian medicine, *Pugionium cornutum* L. Gaertn, Novel bioactive constituents, Thiohydantoin derivatives

## Abstract

The data presented in this article are associated with the research article entitled “**Bioactive Constituents Study of *Pugionium cornutum* L. Gaertn on Intestinal Motility**” [1]. The aim of this data was to provide the 1D, 2D NMR and MS spectrum of novel bioactive compounds from *P. cornutum*.

**Table:** Specifications Table

Subject	Chemistry
Specific subject area	Natural products research
Type of data	Figure
How data were acquired	First, the compounds were isolated from the 70% ethanol-water extract of *P. cornutum* roots. Then the samples were dissolved in DMSO‑*d*_6_ or CD_3_OD before NMR test.
Data format	Raw and Analyzed
Parameters for data collection	The NMR spectra data parameters including chemical shift *δ*-values and coupling constant (*J*). The MS spectra data parameters including *m/z*.
Description of data collection	NMR spectra data of novel compounds from the roots of *P. cornutum* were recorded on a Bruker Advance-500 spectrometer using standard Bruker pulse programs (Bruker, Karlsruhe, Germany). Chemical shifts were shown as *δ*-values with reference to tetramethylsilane (TMS) as an internal standard. Positive- and negative-ion HRESI-TOF-MS of novel compounds from *P. cornutum* roots were recorded on an Agilent Technologies 6520 Accurate-Mass Q-Tof LC/MS spectrometer.
Data source location	Tianjin State Key Laboratory of Modern Chinese Medicine, Tianjin, China
Data accessibility	Data is with the article [1]
Related research article	W. Shi, J. Ruan, Y. Guo, Z. Ding, J. Yan, L. Qu, C. Zheng, Y. Zhang, T. Wang, Bioactive constituents study of Pugionium cornutum L. Gaertn on intestinal motility, Fitoerapia, 138 (2019) 104291.

**Table:** 

**Value of the Data** • NMR and MS data of novel compounds is important for elucidating their chemical structures. • NMR and MS data of novel thiohydantoin derivatives is useful for the elucidating their chemical analogues. • This information will allow comparisons across different thiohydantoin derivatives and other new compounds from *Pugionium* species or Cruciferae family plant sources.

## 1 Data

The dataset contains raw analysis data obtained through the chemistry research of the dried roots of Mongolian medicinal and edible plant, *Pugionium cornutum* L. Gaertn. Information about the eight new compounds pugcornols A (**1**), B (**2**), C (**3**), and D (**4**), pugcornosides A (**5**), B (**6**), C (**7**), and D (**8**) has been presented in Fig. 1, Fig. 2, Fig. 3, Fig. 4, Fig. 5, Fig. 6, Fig. 7, Fig. 8, Fig. 9, Fig. 10, Fig. 11, Fig. 12, Fig. 13, Fig. 14, Fig. 15, Fig. 16, Fig. 17, Fig. 18, Fig. 19, Fig. 20, Fig. 21, Fig. 22, Fig. 23, Fig. 24, Fig. 25, Fig. 26, Fig. 27, Fig. 28, Fig. 29, Fig. 30, Fig. 31, Fig. 32, Fig. 33, Fig. 34, Fig. 35, Fig. 36, Fig. 37, Fig. 38, Fig. 39, Fig. 40, Fig. 41, Fig. 42, Fig. 43, Fig. 44, Fig. 45.

**Fig. 1:**
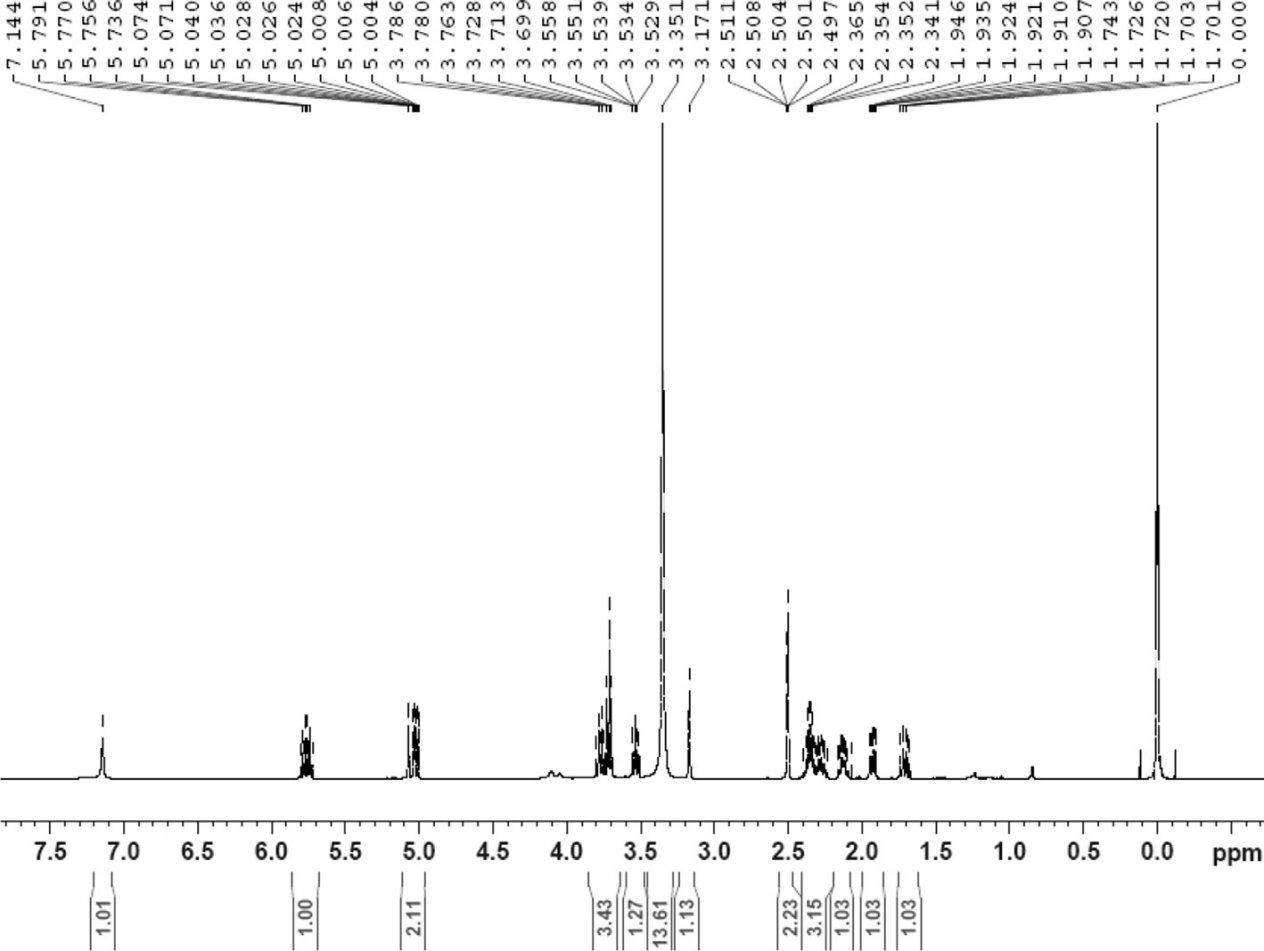
^1^H NMR (500 MHz, DMSO‑*d*_6_) spectrum of compound **1**.

**Fig. 2:**
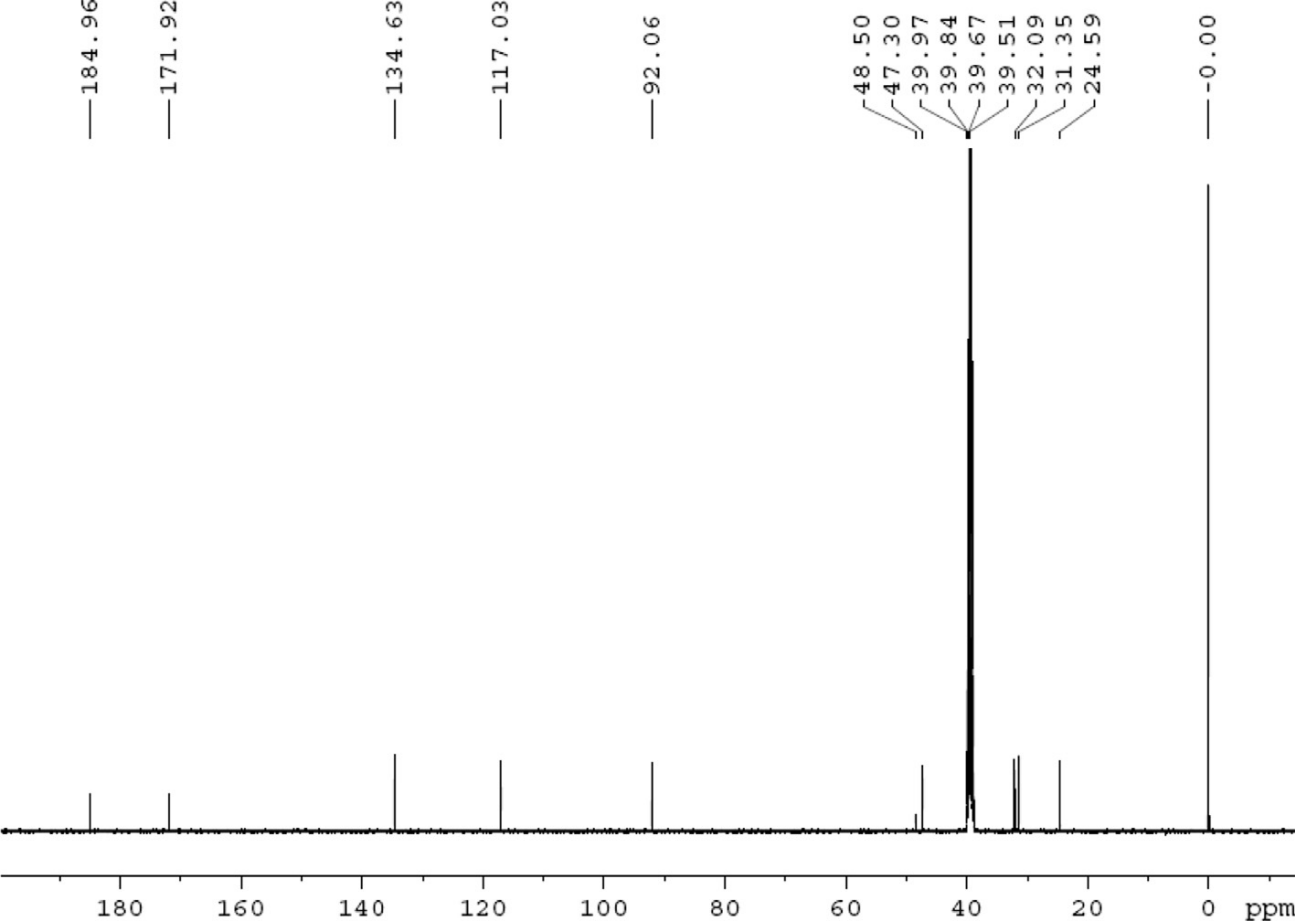
^13^C NMR (125 MHz, DMSO‑*d*_6_) spectrum of compound **1**.

**Fig. 3:**
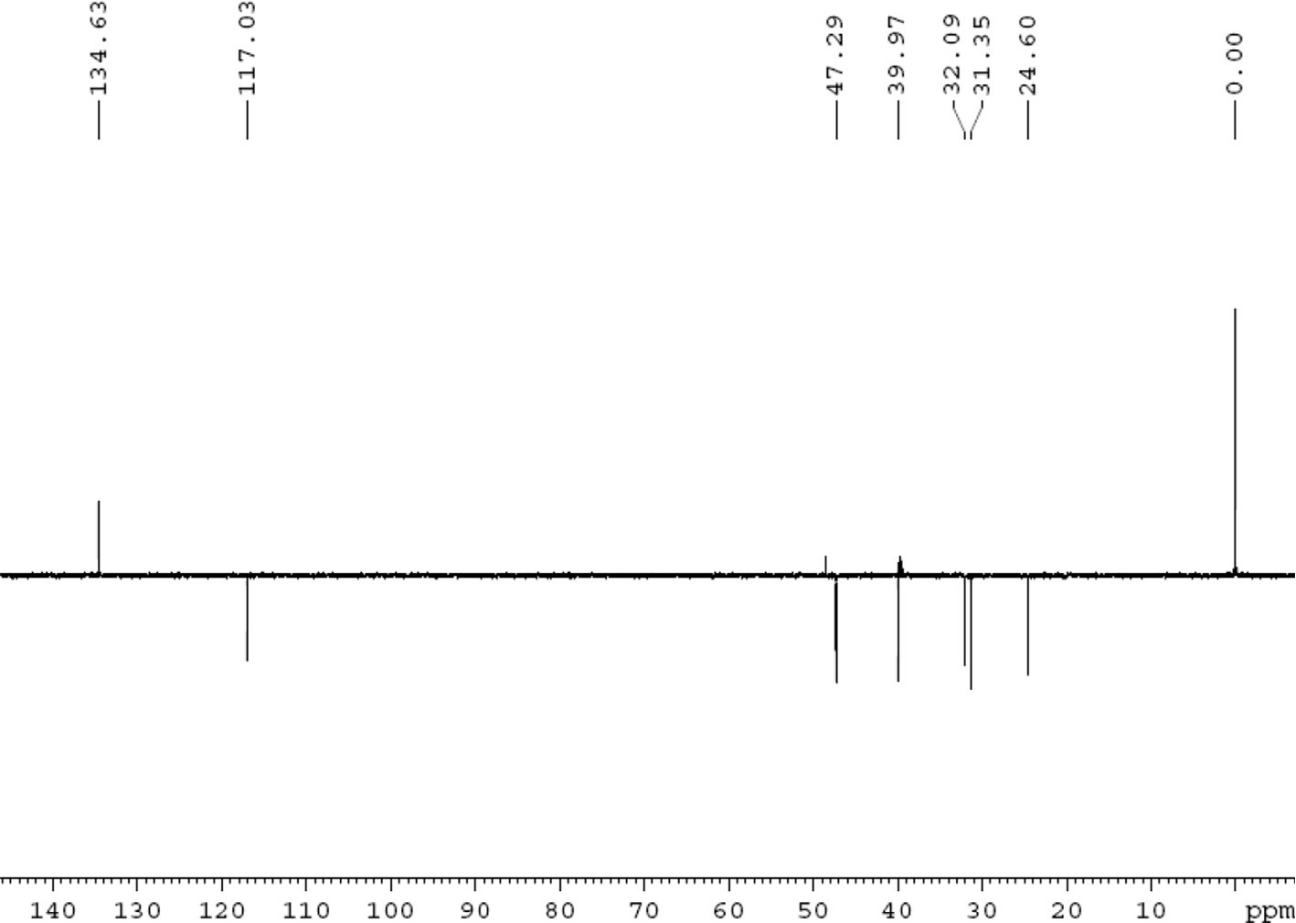
DEPT 135 (DMSO‑*d*_6_) spectrum of compound **1**.

**Fig. 4:**
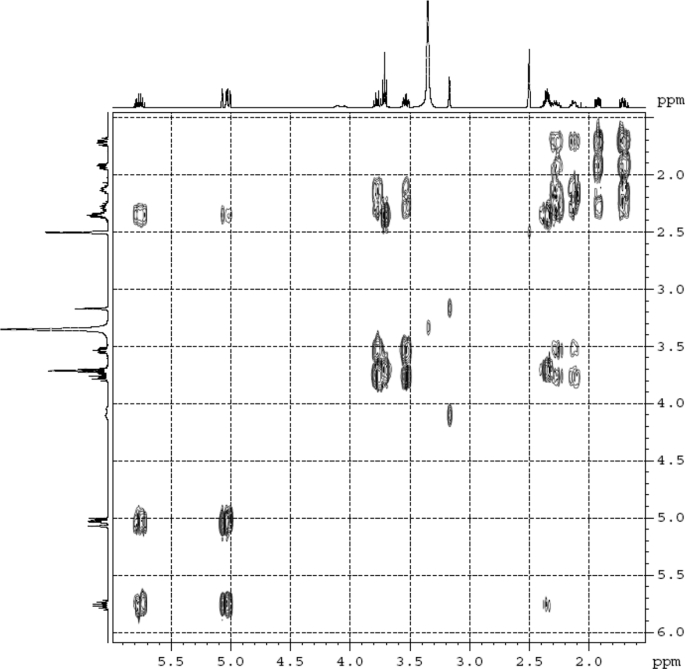
^1^H ^1^H COSY (DMSO‑*d*_6_) spectrum of compound **1**.

**Fig. 5:**
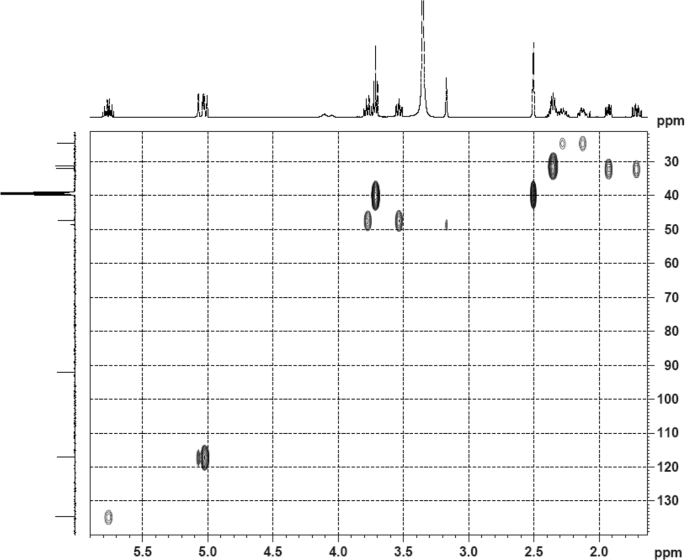
HSQC (DMSO‑*d*_6_) spectrum of compound **1**.

**Fig. 6:**
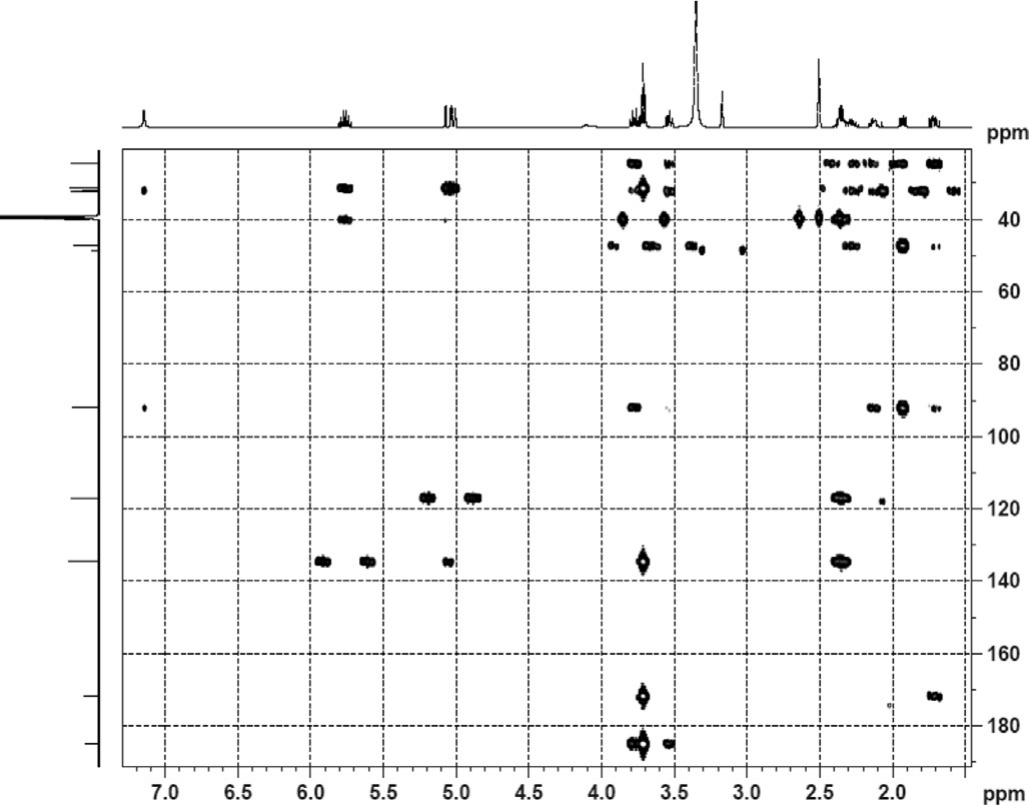
HMBC (DMSO‑*d*_6_) spectrum of compound **1**.

**Fig. 7:**
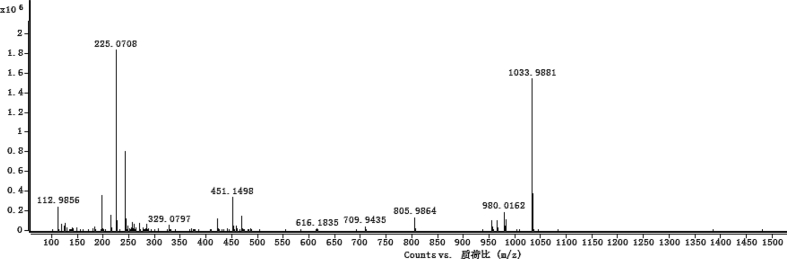
HRESI-TOF-MS spectrum of compound **1**.

**Fig. 8:**
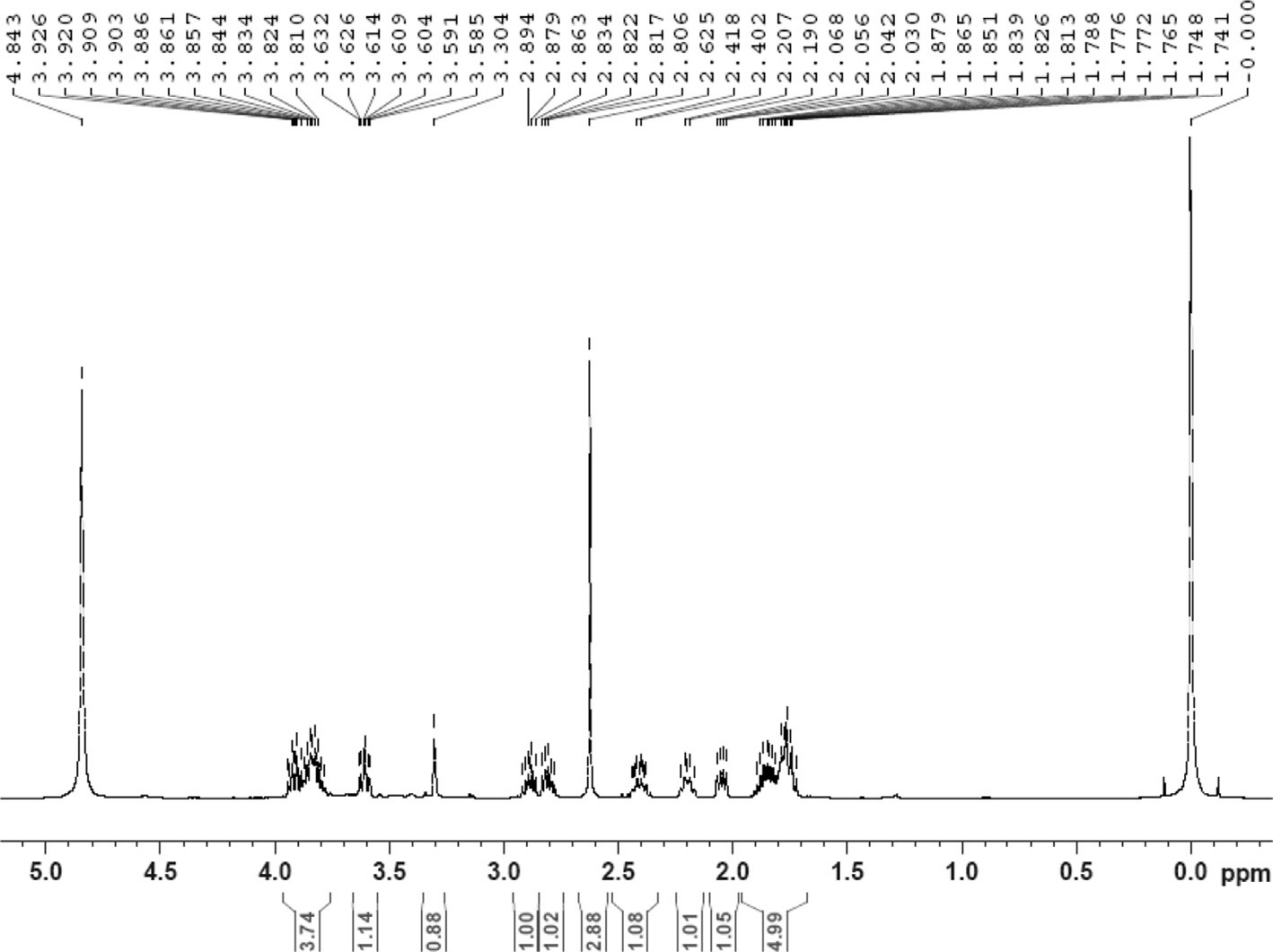
^1^H NMR (500 MHz, CD_3_OD) spectrum of compound **2**.

**Fig. 9:**
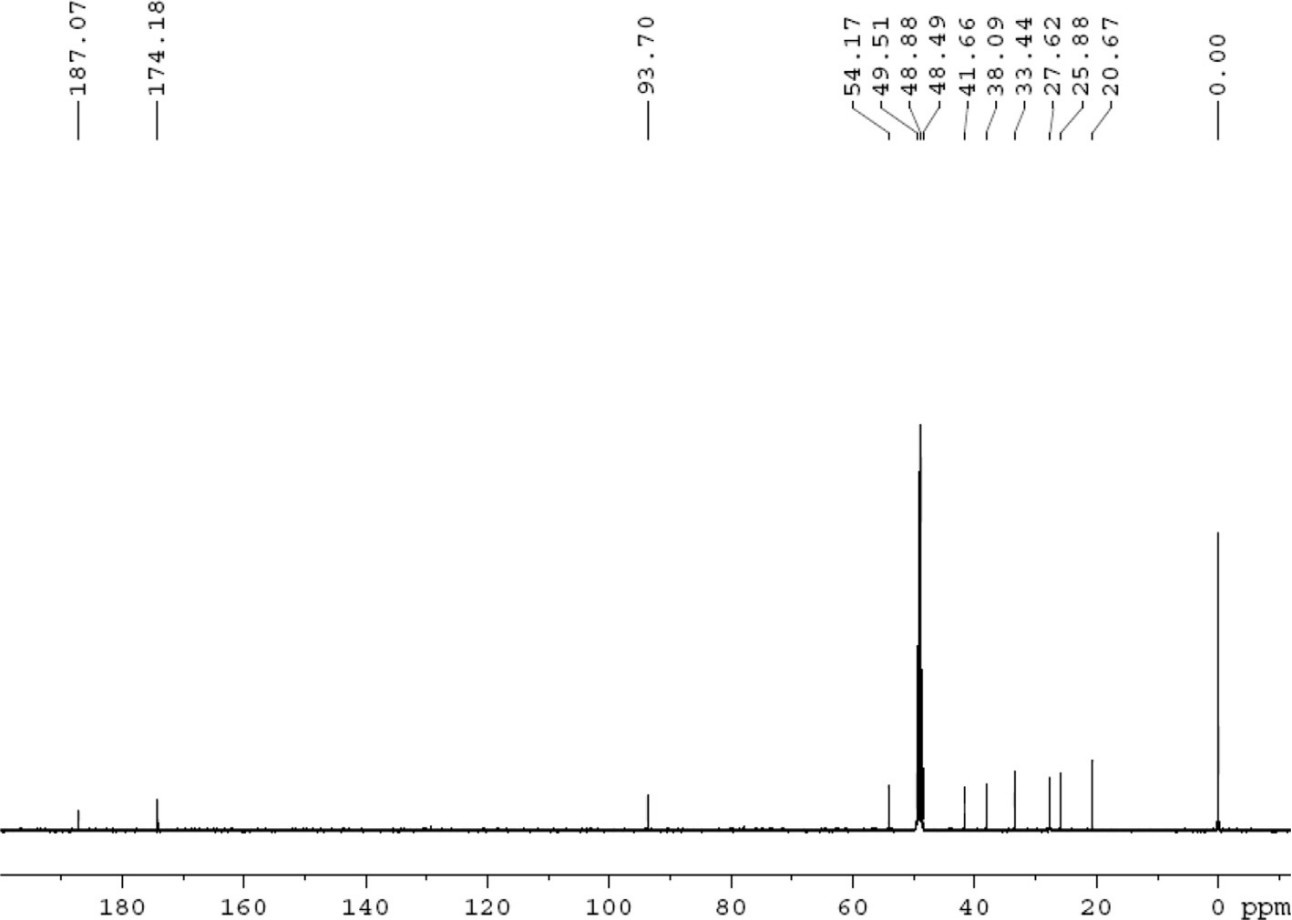
^13^C NMR (125 MHz, CD_3_OD) spectrum of compound **2**.

**Fig. 10:**
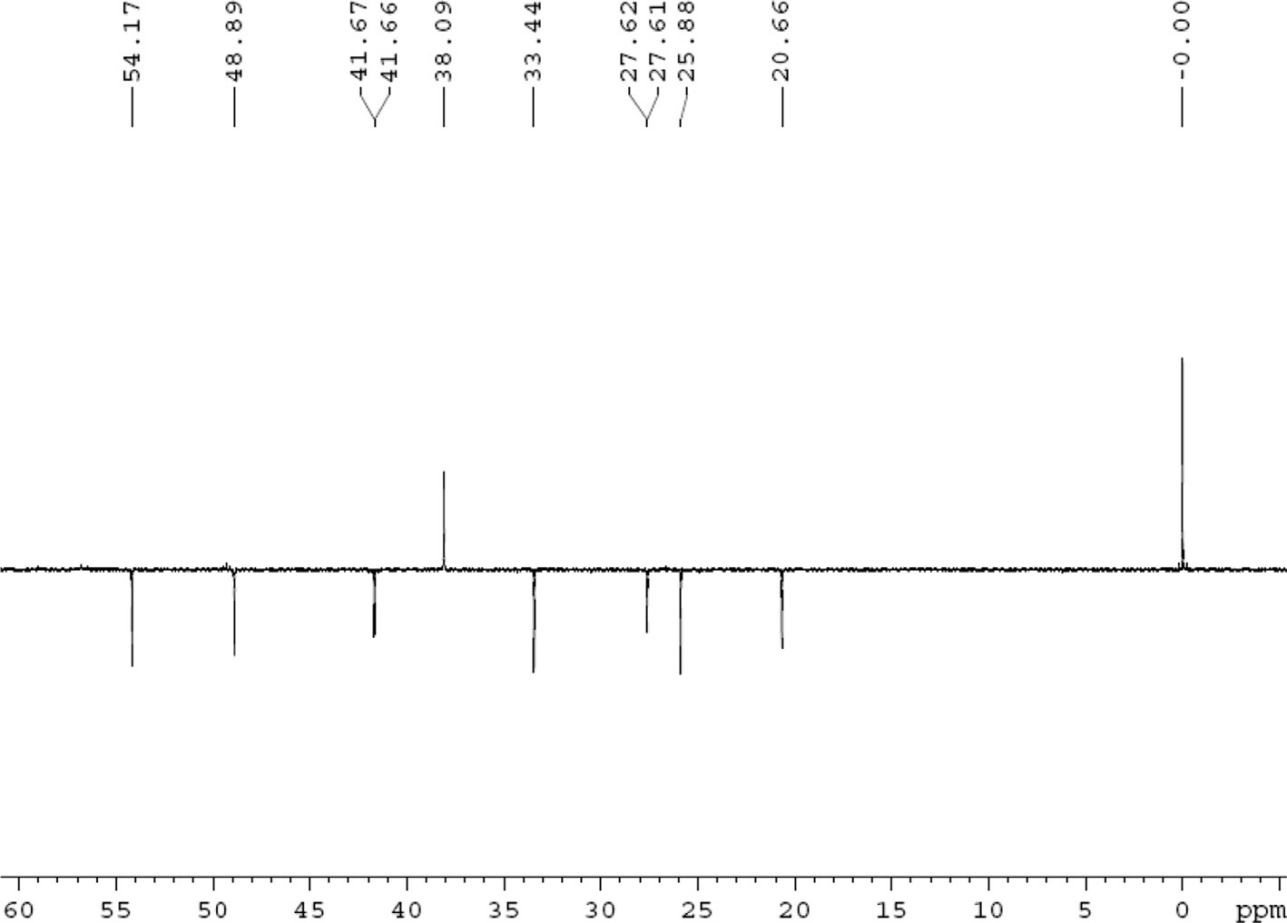
DEPT 135 (CD_3_OD) spectrum of compound **2**.

**Fig. 11:**
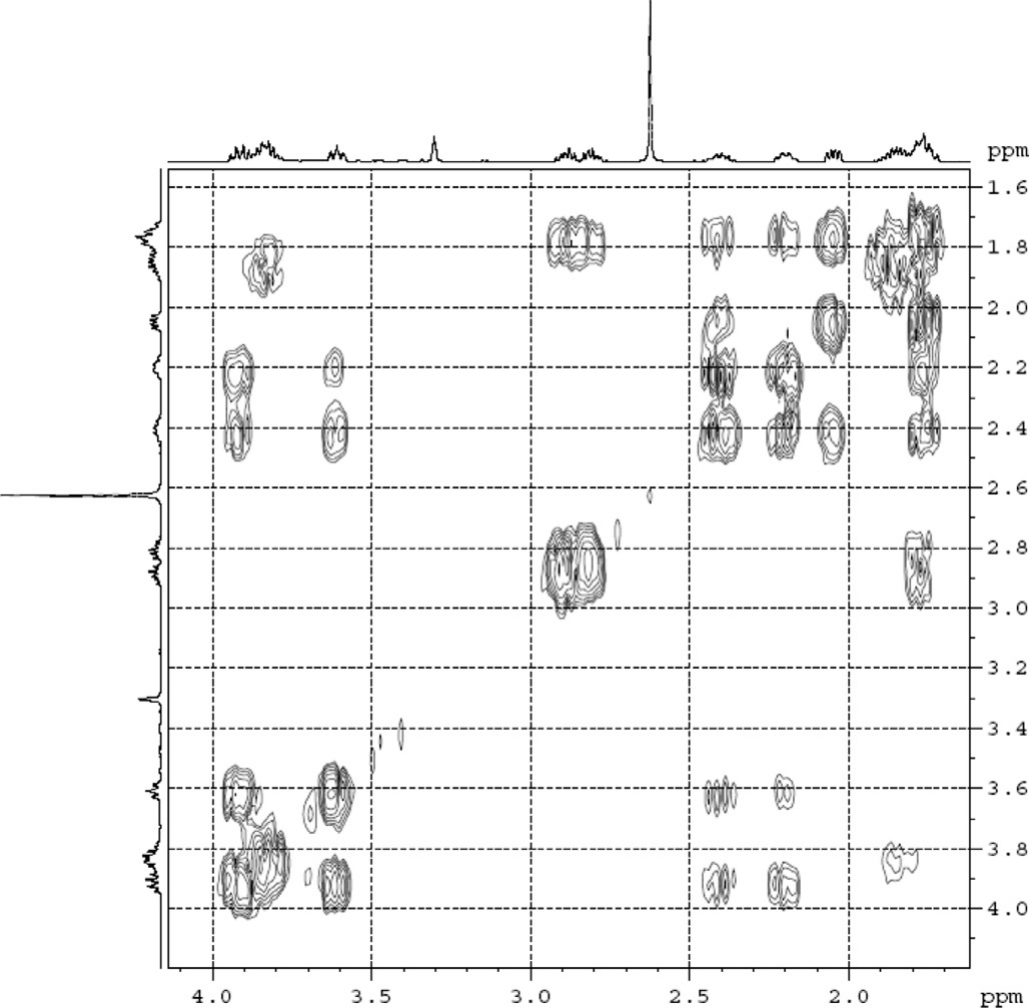
^1^H ^1^H COSY (CD_3_OD) spectrum of compound **2**.

**Fig. 12:**
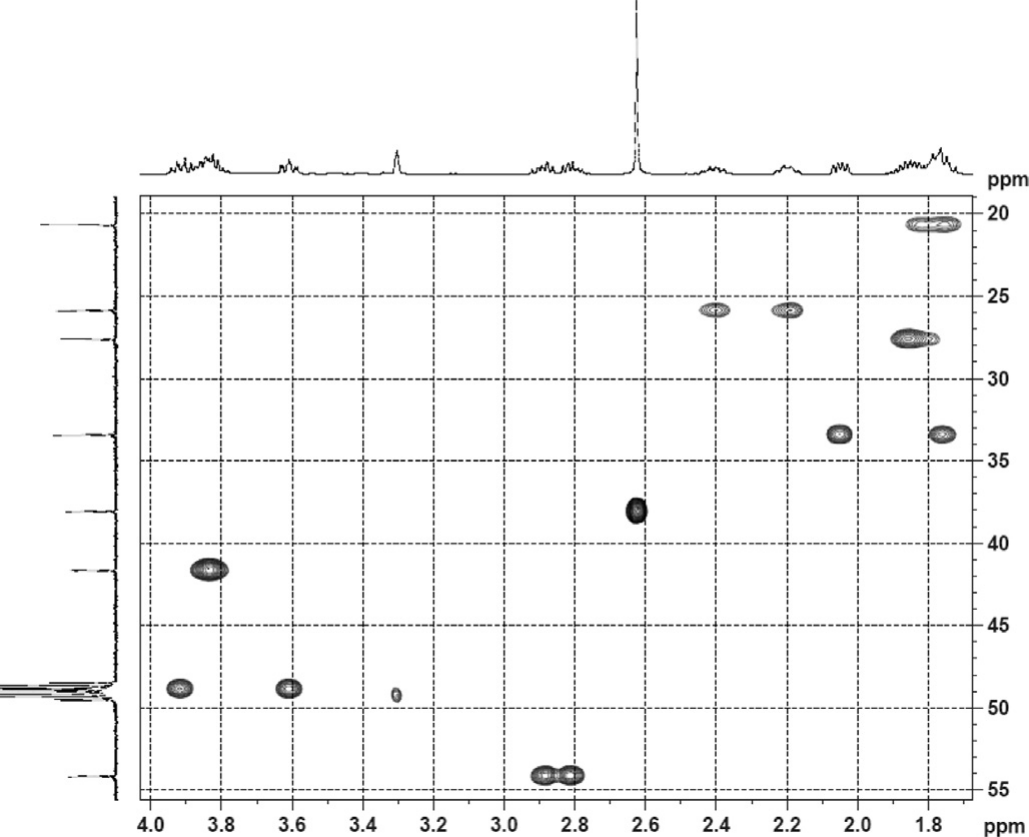
HSQC (CD_3_OD) spectrum of compound **2**.

**Fig. 13:**
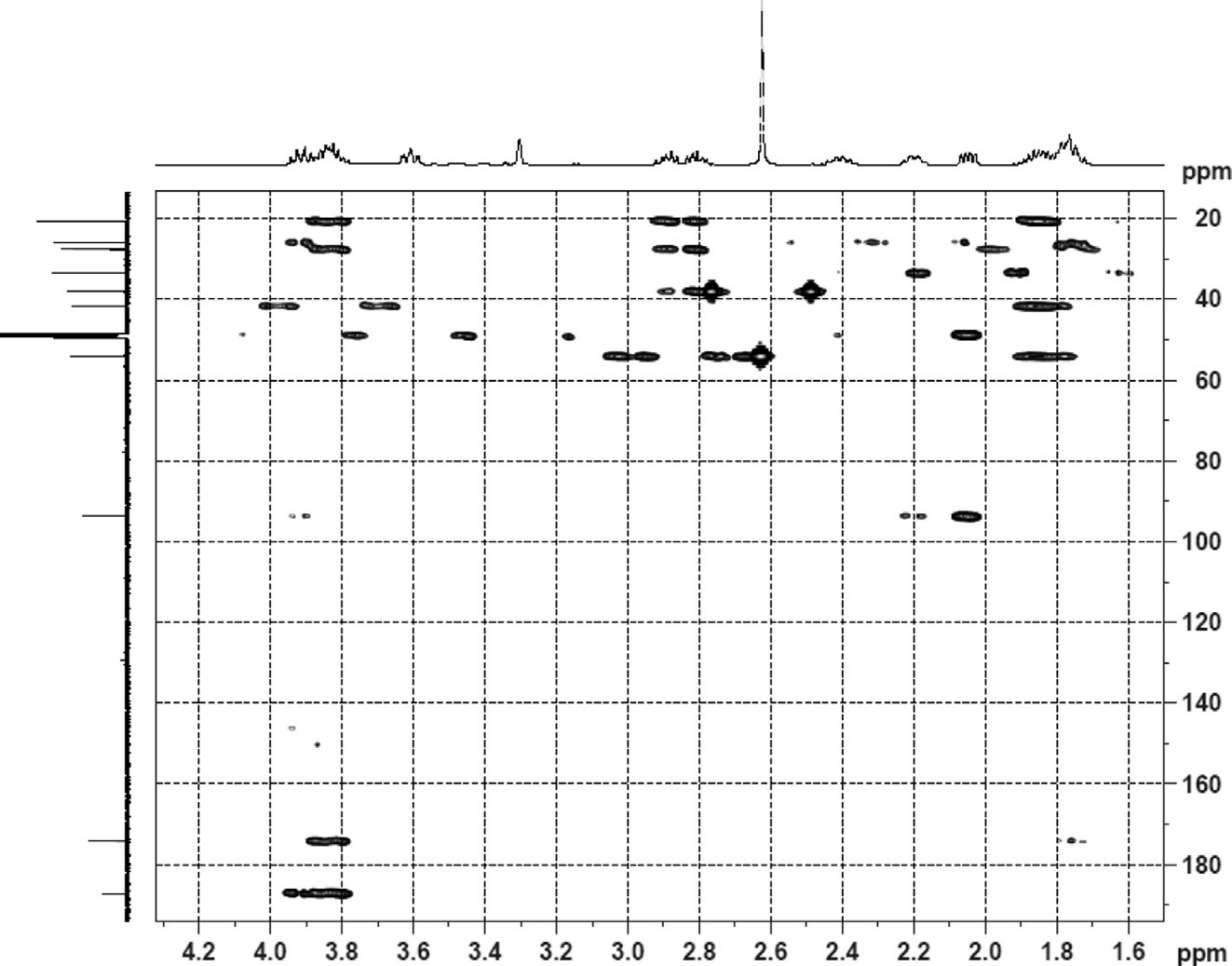
HMBC (CD_3_OD) spectrum of compound **2**.

**Fig. 14:**
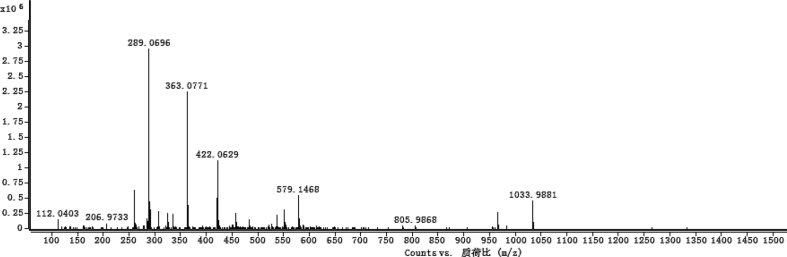
HRESI-TOF-MS spectrum of compound **2**.

**Fig. 15:**
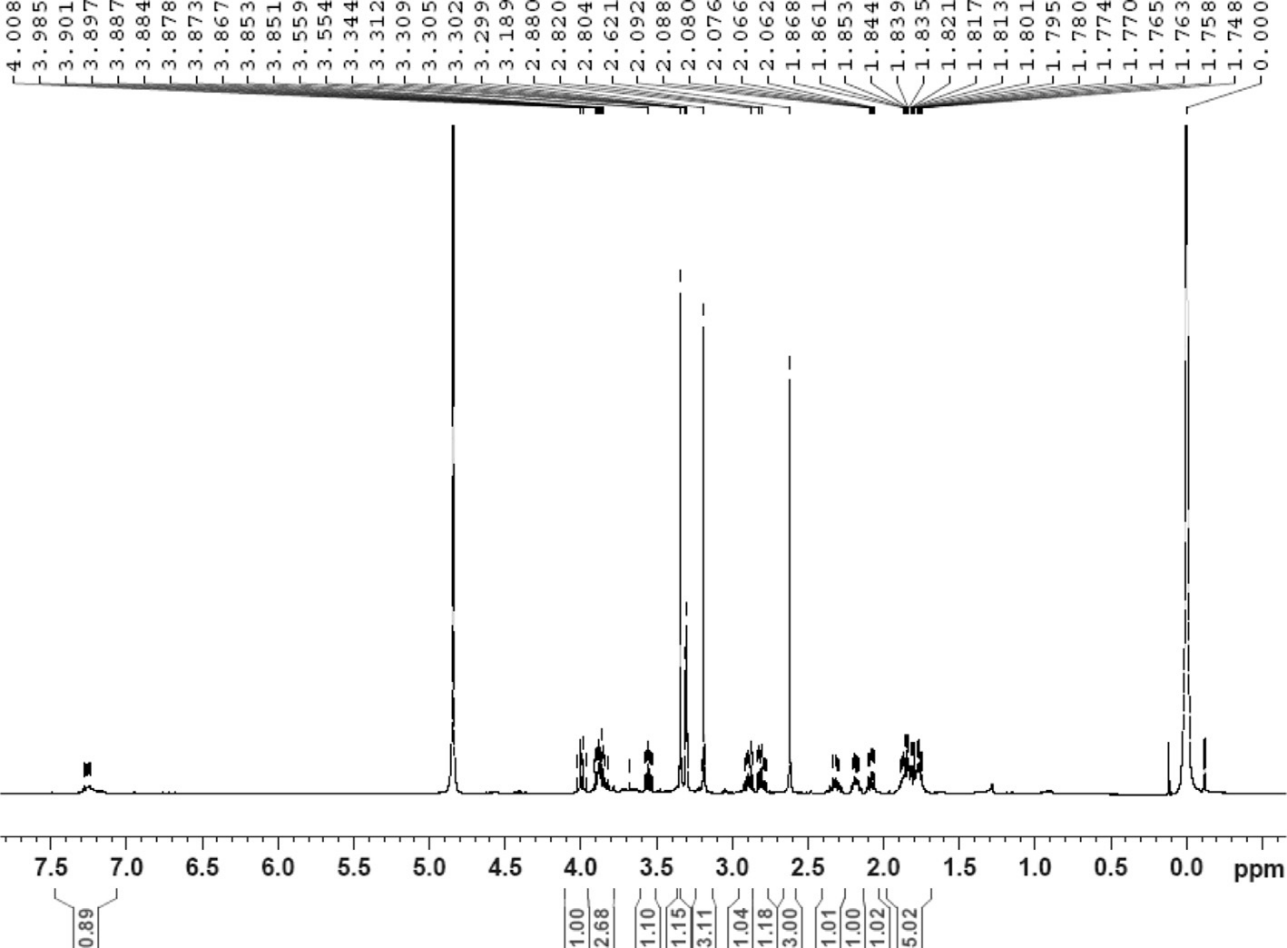
^1^H NMR (500 MHz, CD_3_OD) spectrum of compound **3**.

**Fig. 16:**
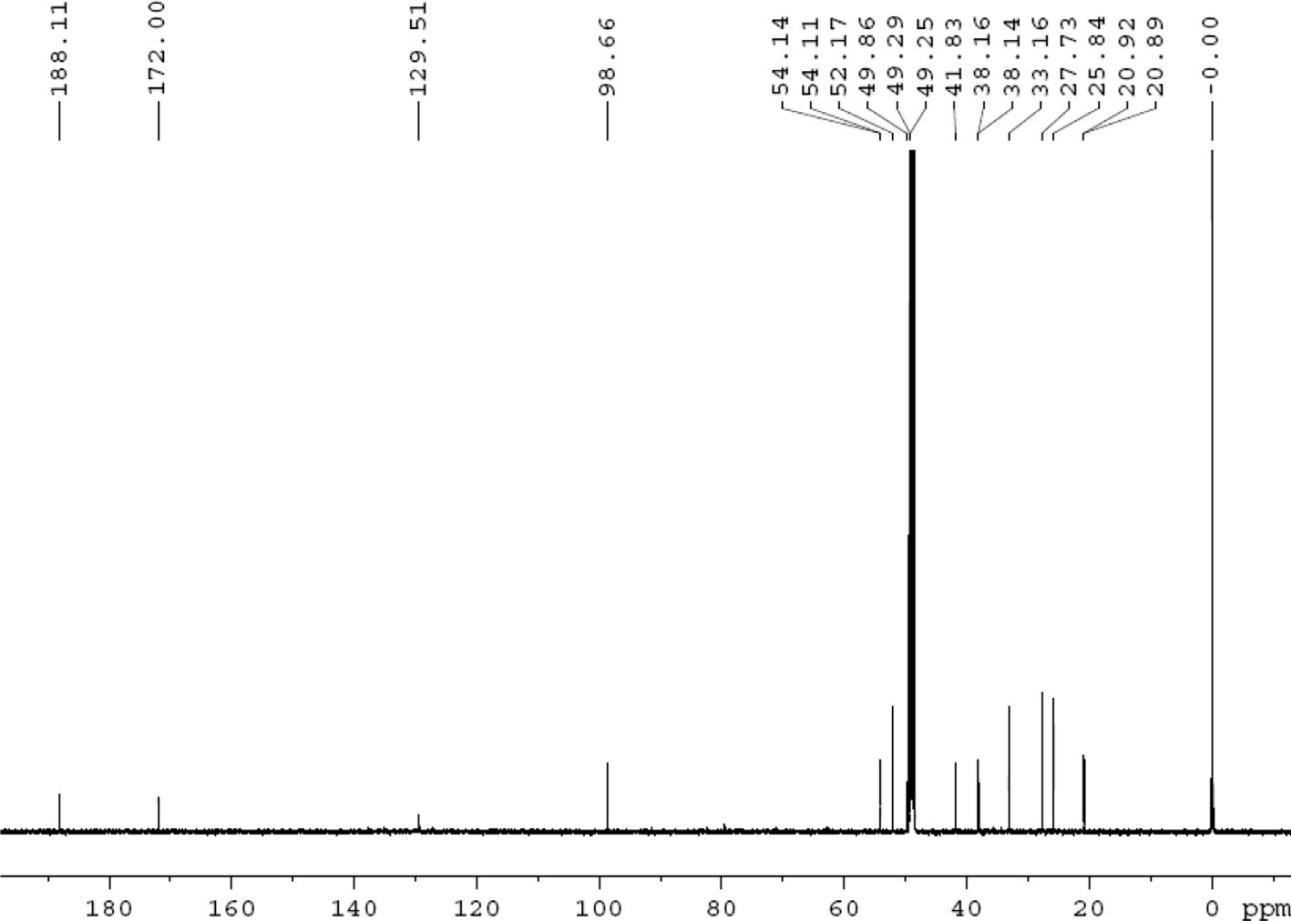
^13^C NMR (125 MHz, CD_3_OD) spectrum of compound **3**.

**Fig. 17:**
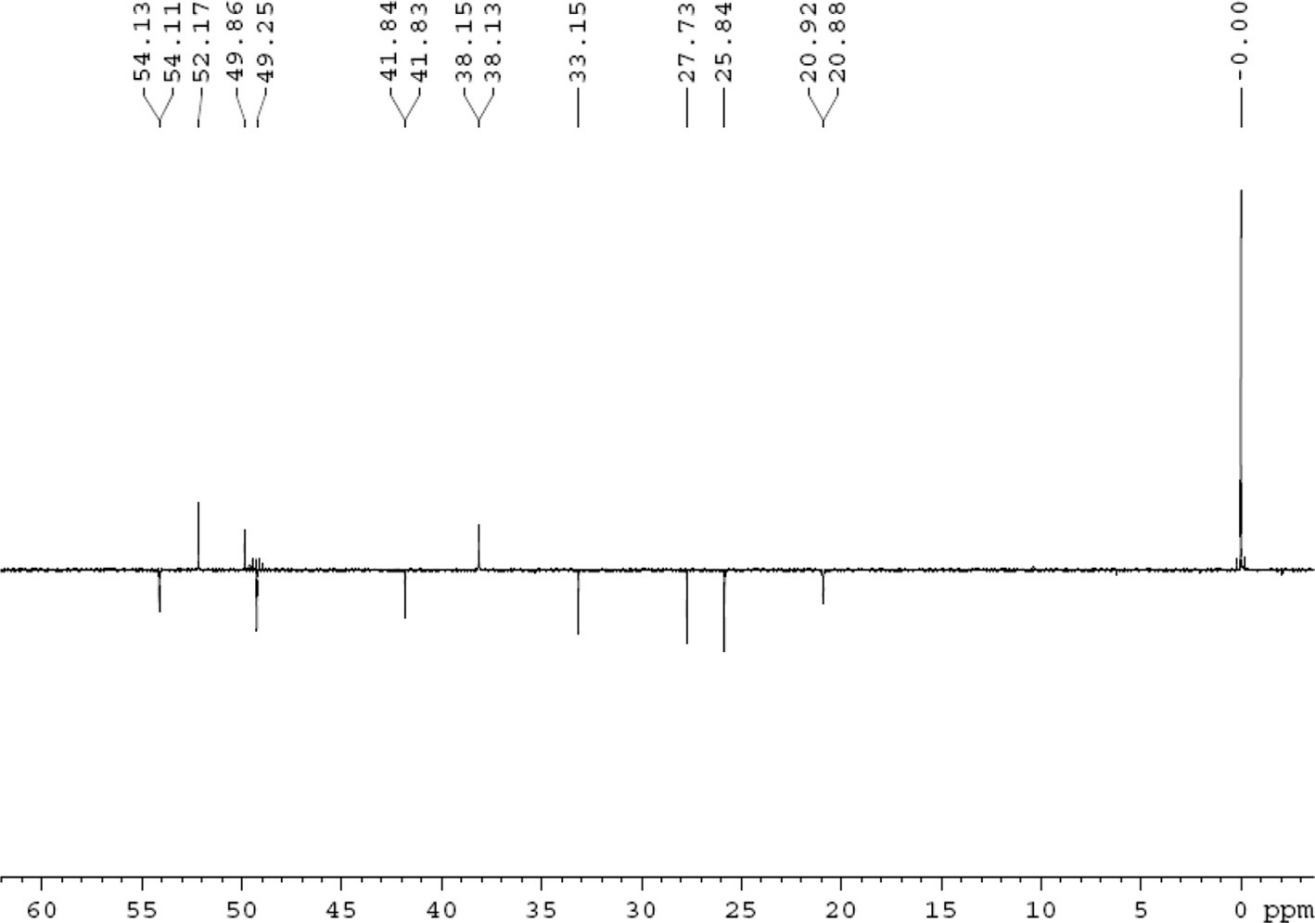
DEPT 135 (CD_3_OD) spectrum of compound **3**.

**Fig. 18:**
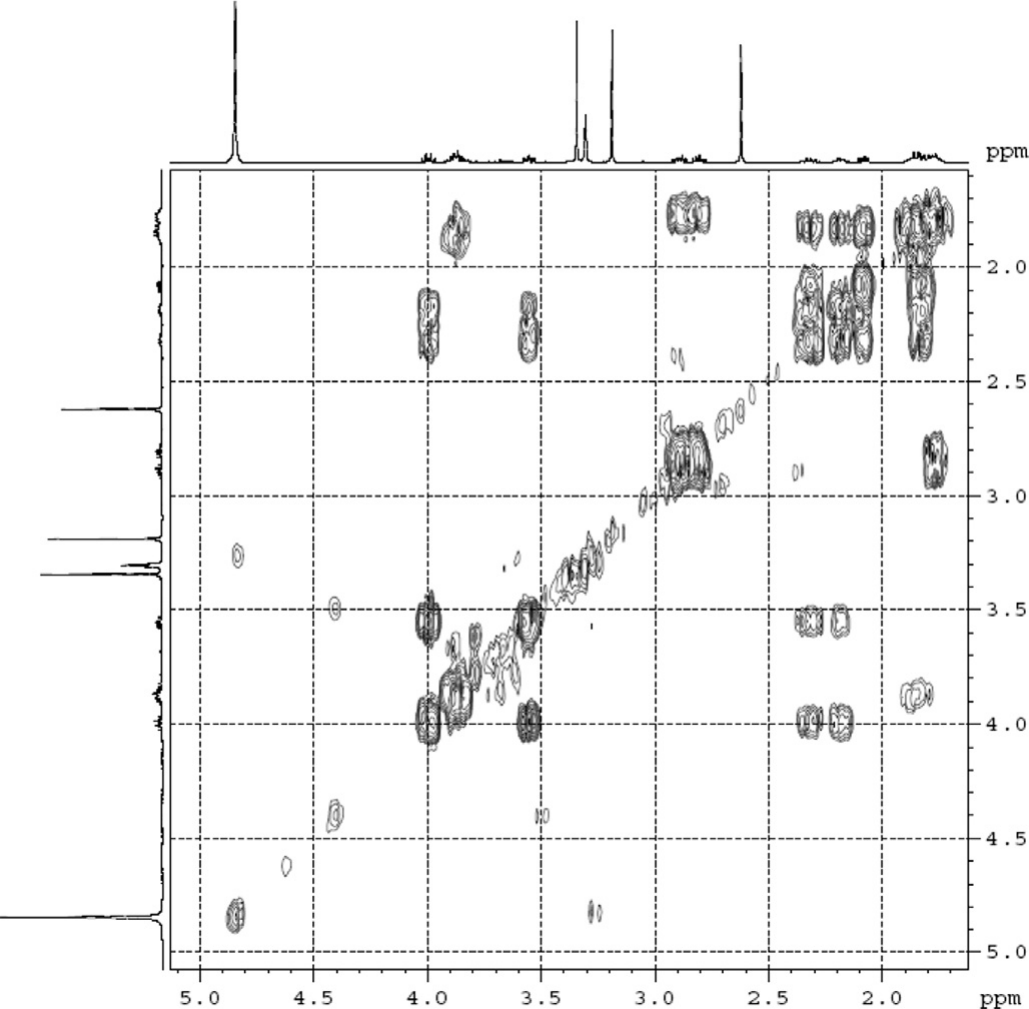
^1^H ^1^H COSY (CD_3_OD) spectrum of compound **3**.

**Fig. 19:**
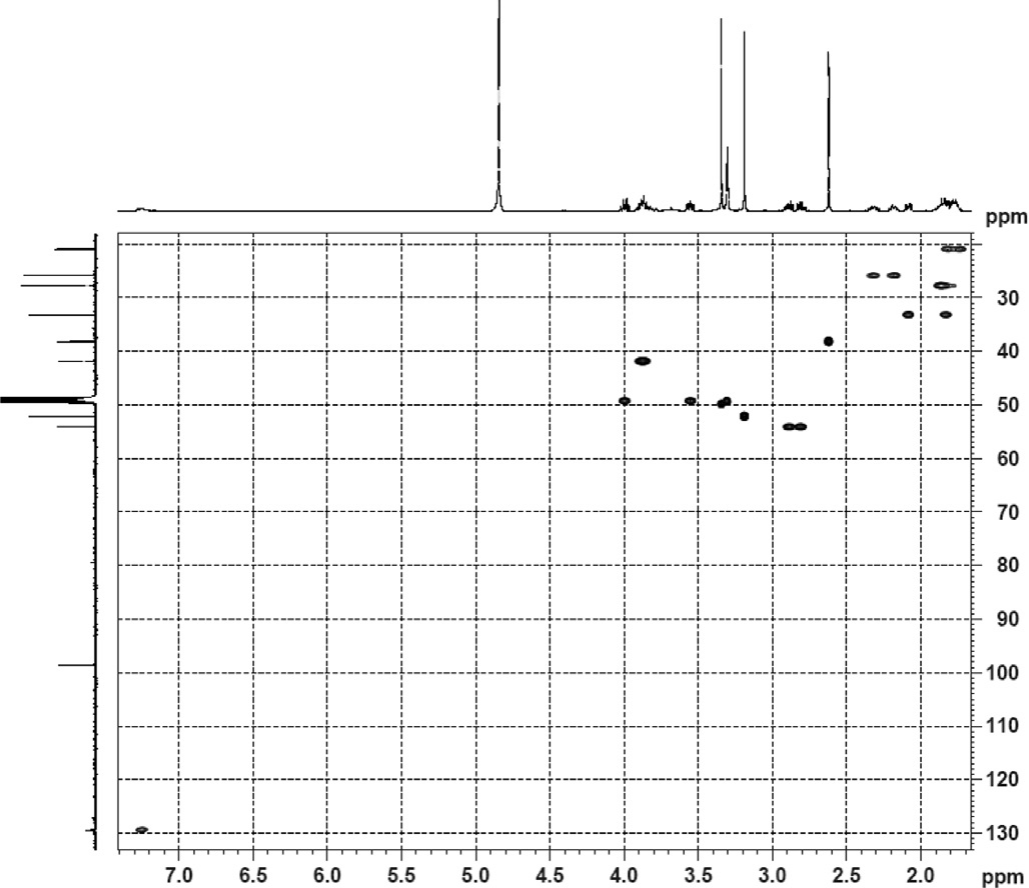
HSQC (CD_3_OD) spectrum of compound **3**.

**Fig. 20:**
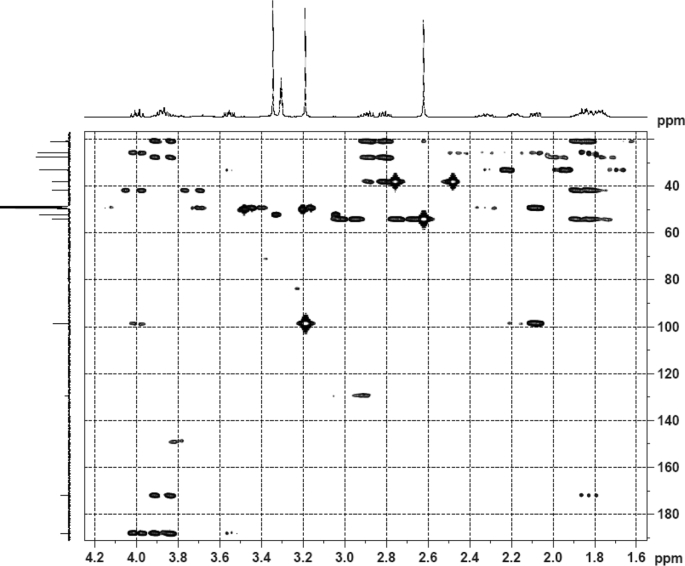
HMBC (CD_3_OD) spectrum of compound **3**.

**Fig. 21:**
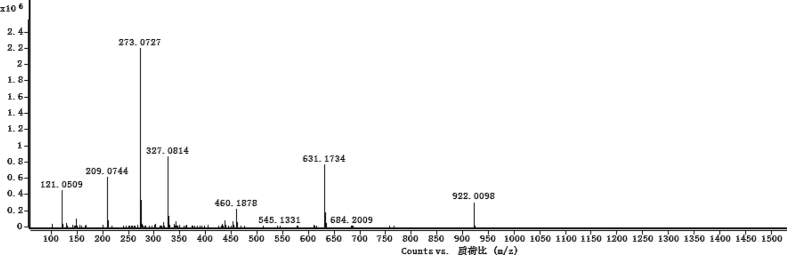
HRESI-TOF-MS spectrum of compound **3**.

**Fig. 22:**
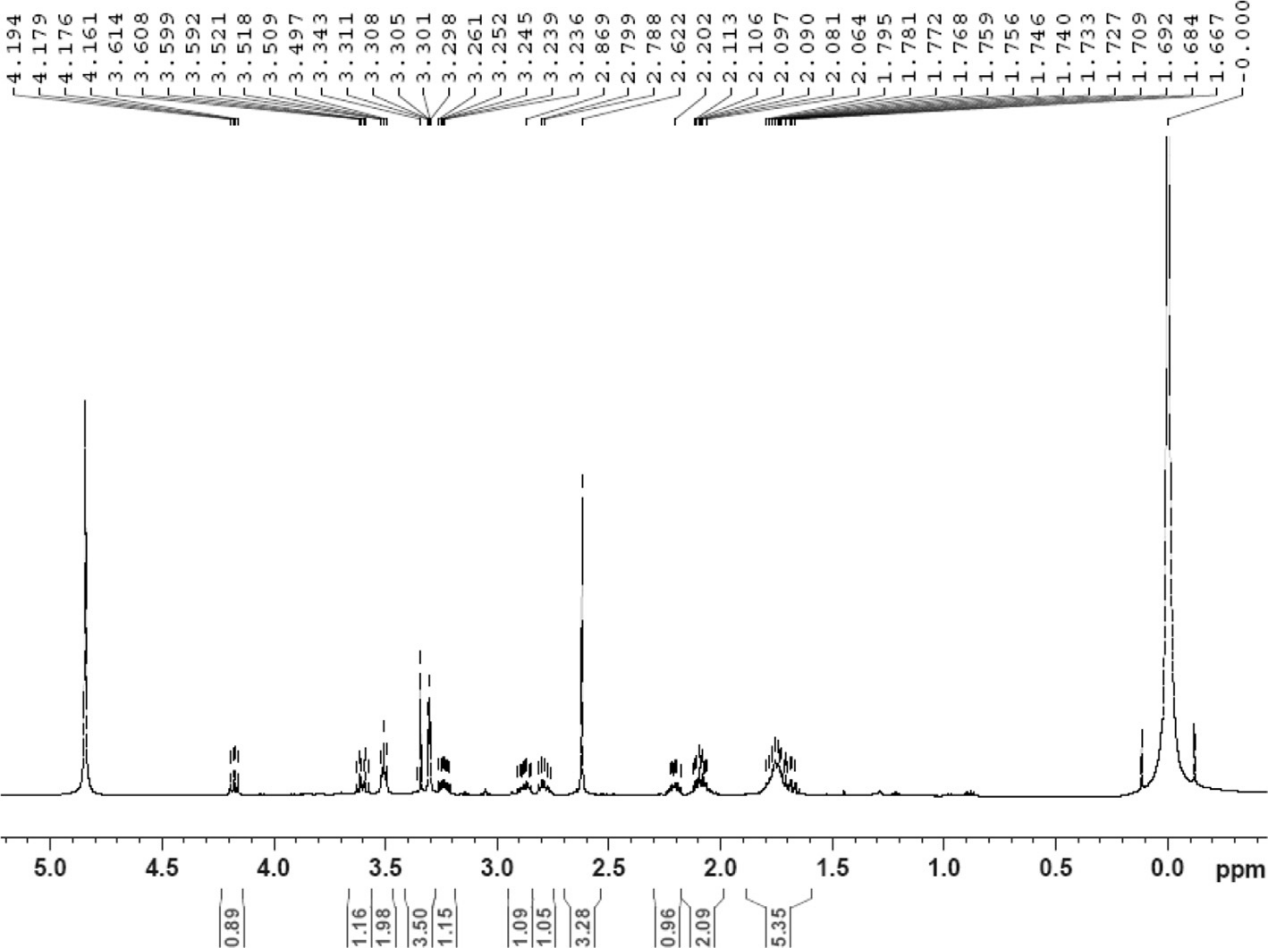
^1^H NMR (500 MHz, CD_3_OD) spectrum of compound **4**.

**Fig. 23:**
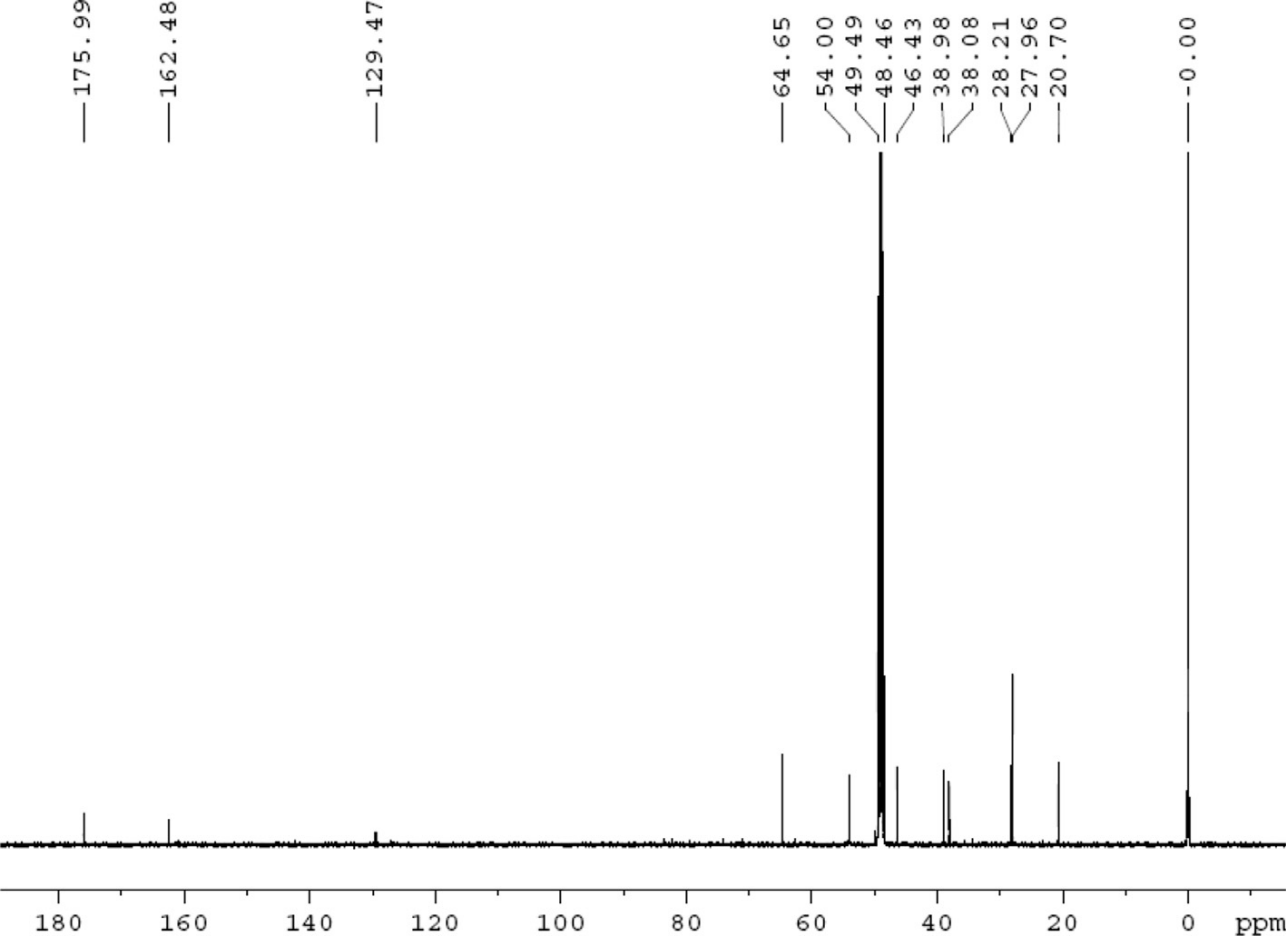
^13^C NMR (125 MHz, CD_3_OD) spectrum of compound **4**.

**Fig. 24:**
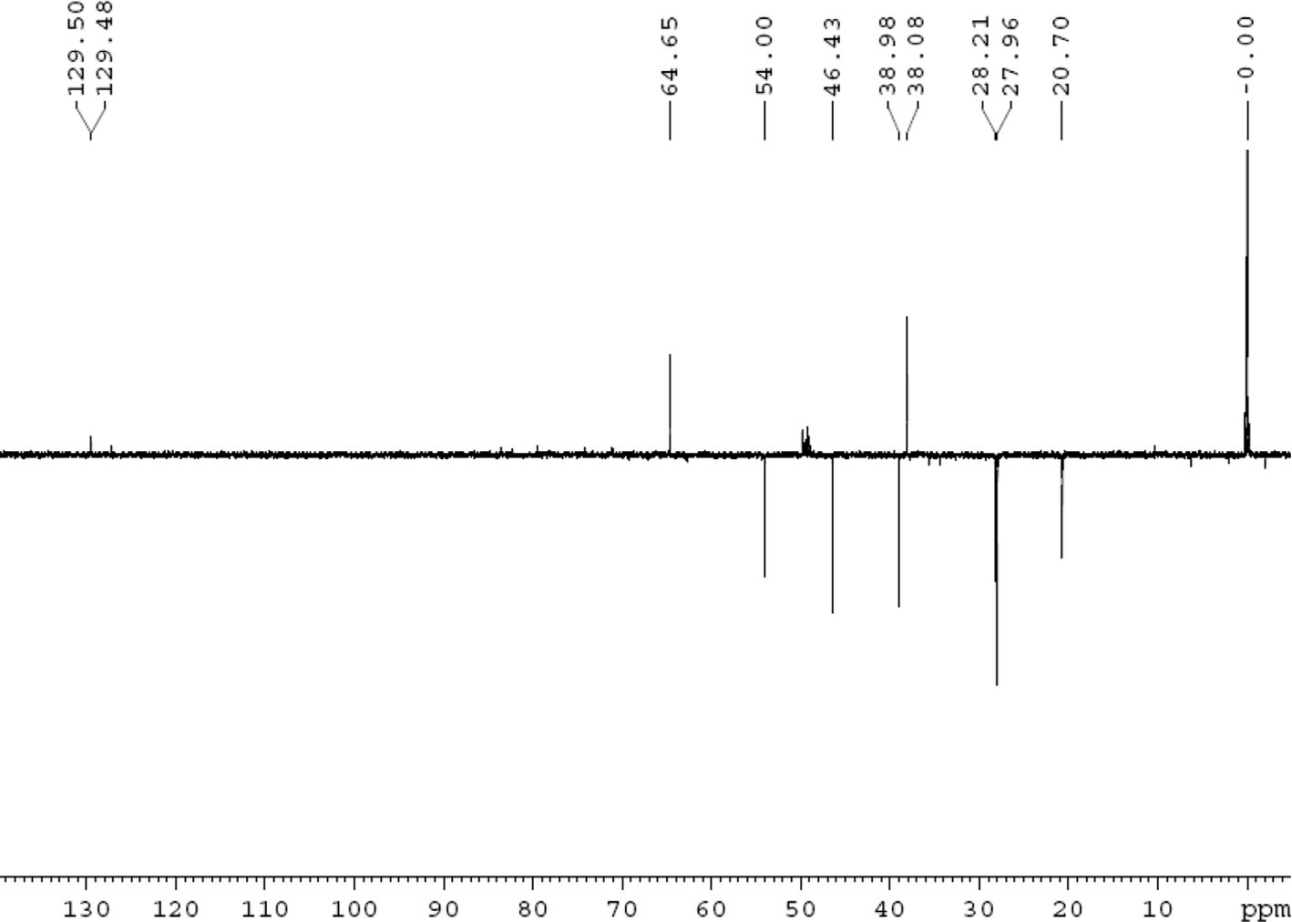
DEPT 135 (CD_3_OD) spectrum of compound **4**.

**Fig. 25:**
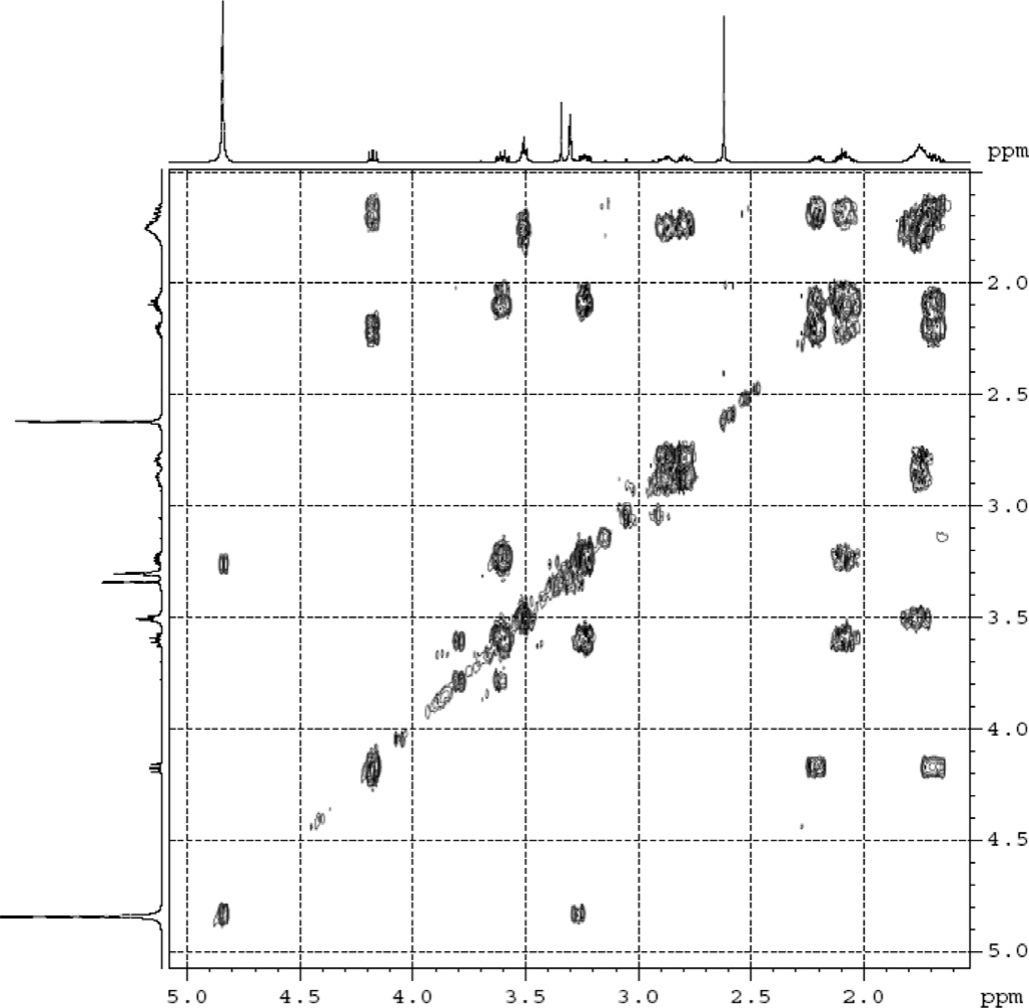
^1^H ^1^H COSY (CD_3_OD) spectrum of compound **4**.

**Fig. 26:**
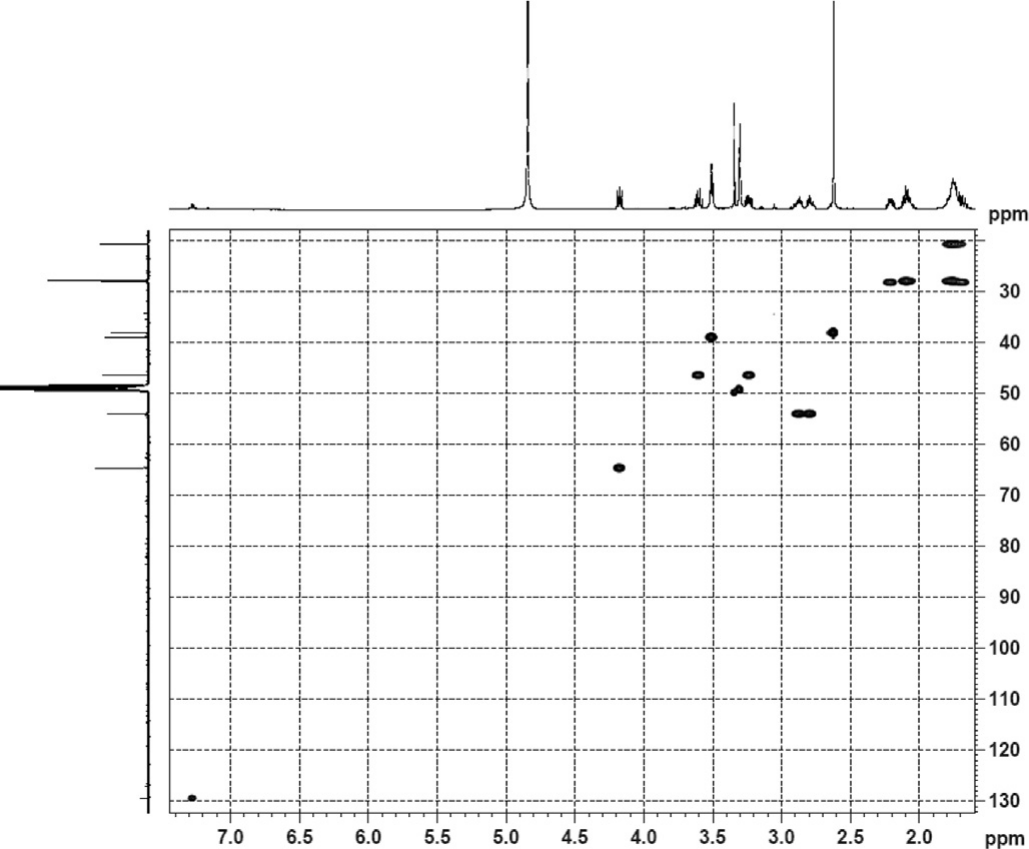
HSQC (CD_3_OD) spectrum of compound **4**.

**Fig. 27:**
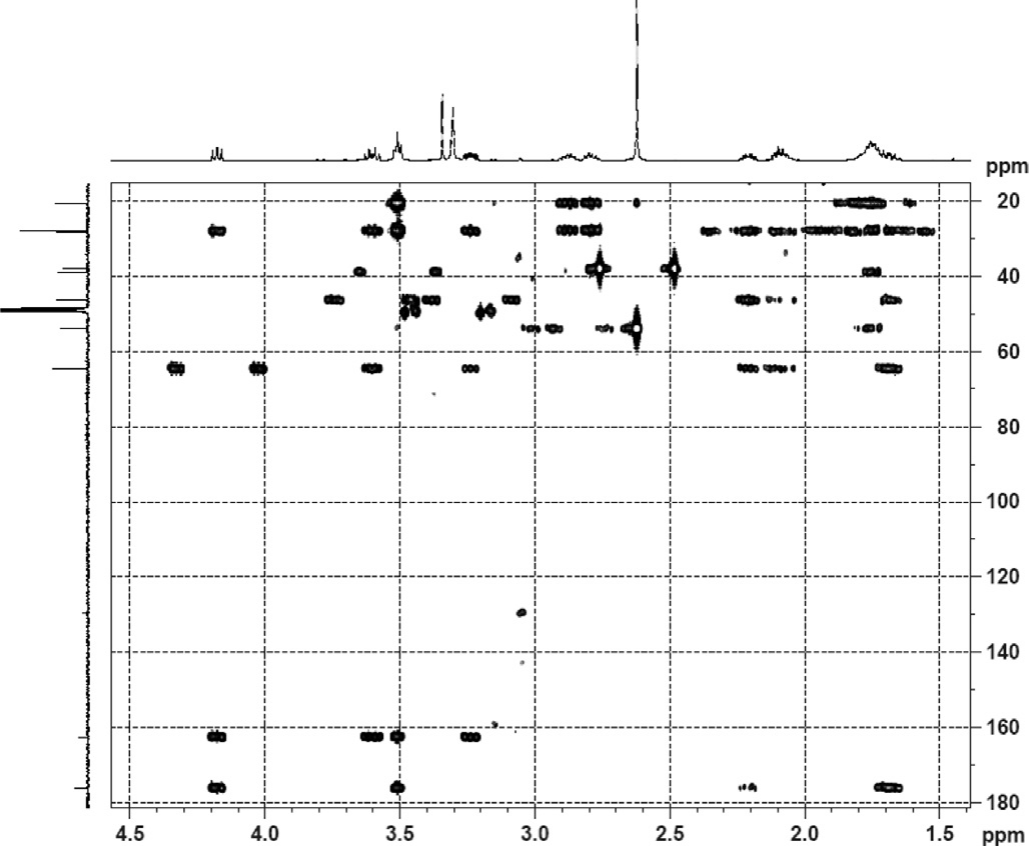
HMBC (CD_3_OD) spectrum of compound **4**.

**Fig. 28:**
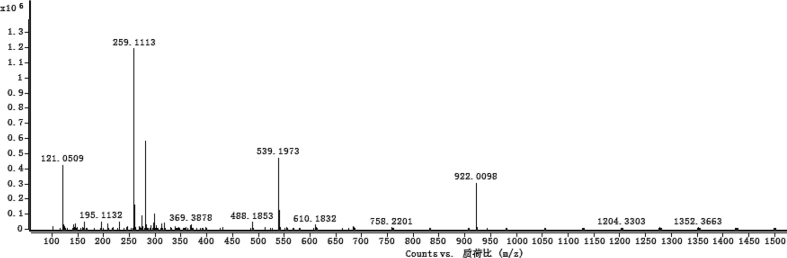
HRESI-TOF-MS spectrum of compound **4**.

**Fig. 29:**
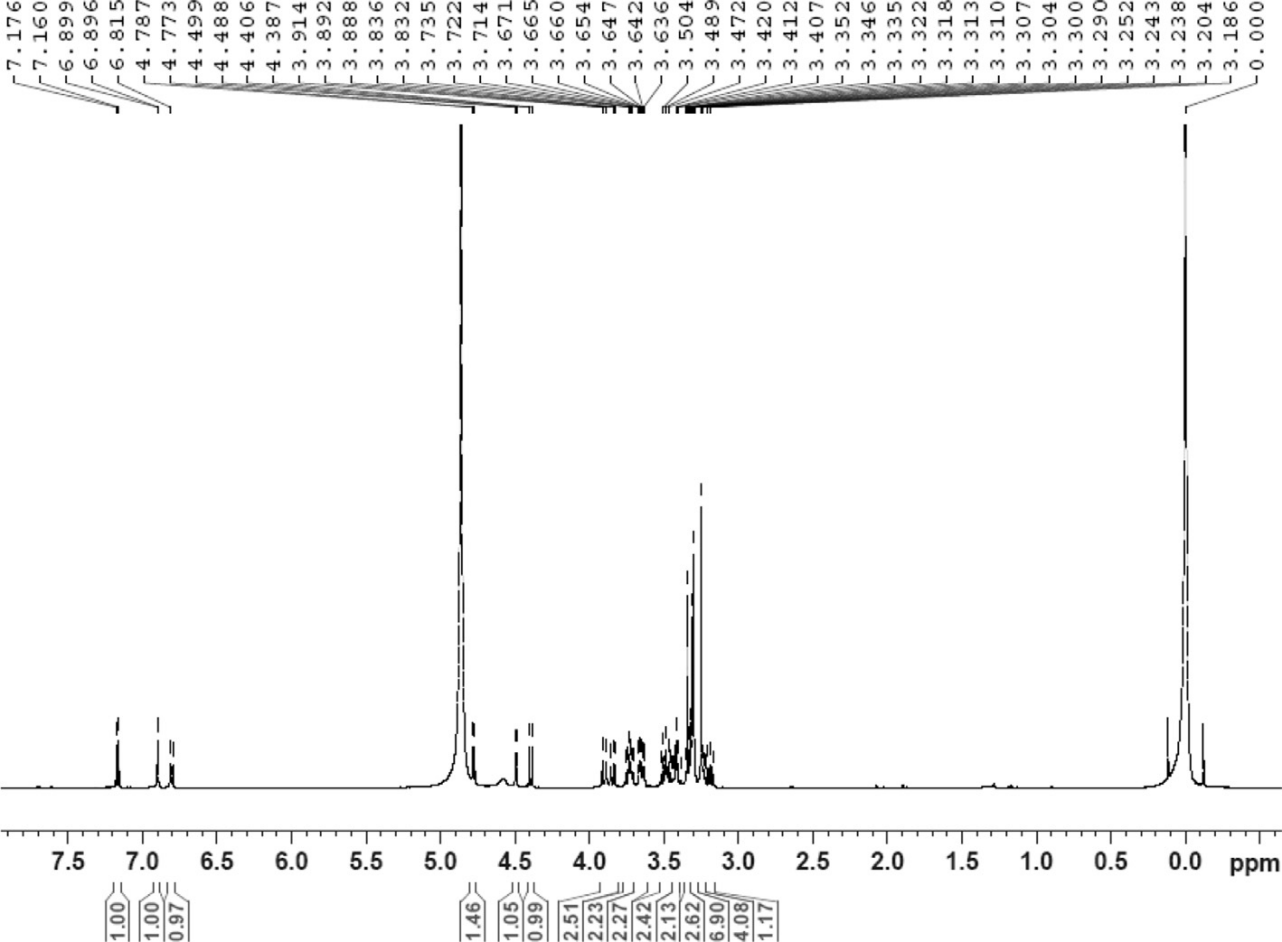
^1^H NMR (500 MHz, CD_3_OD) spectrum of compound **5**.

**Fig. 30:**
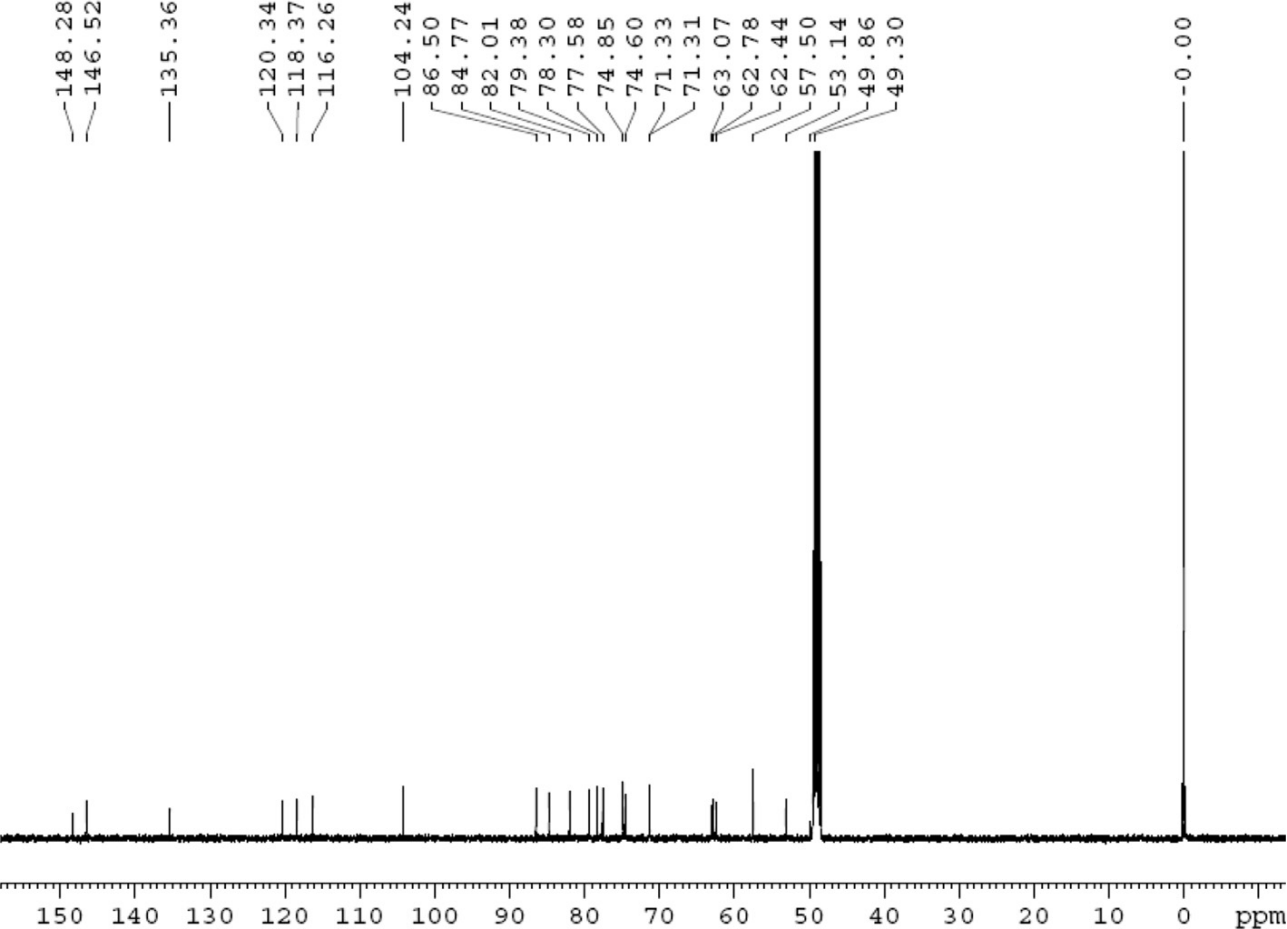
^13^C NMR (125 MHz, CD_3_OD) spectrum of compound **5**.

**Fig. 31:**
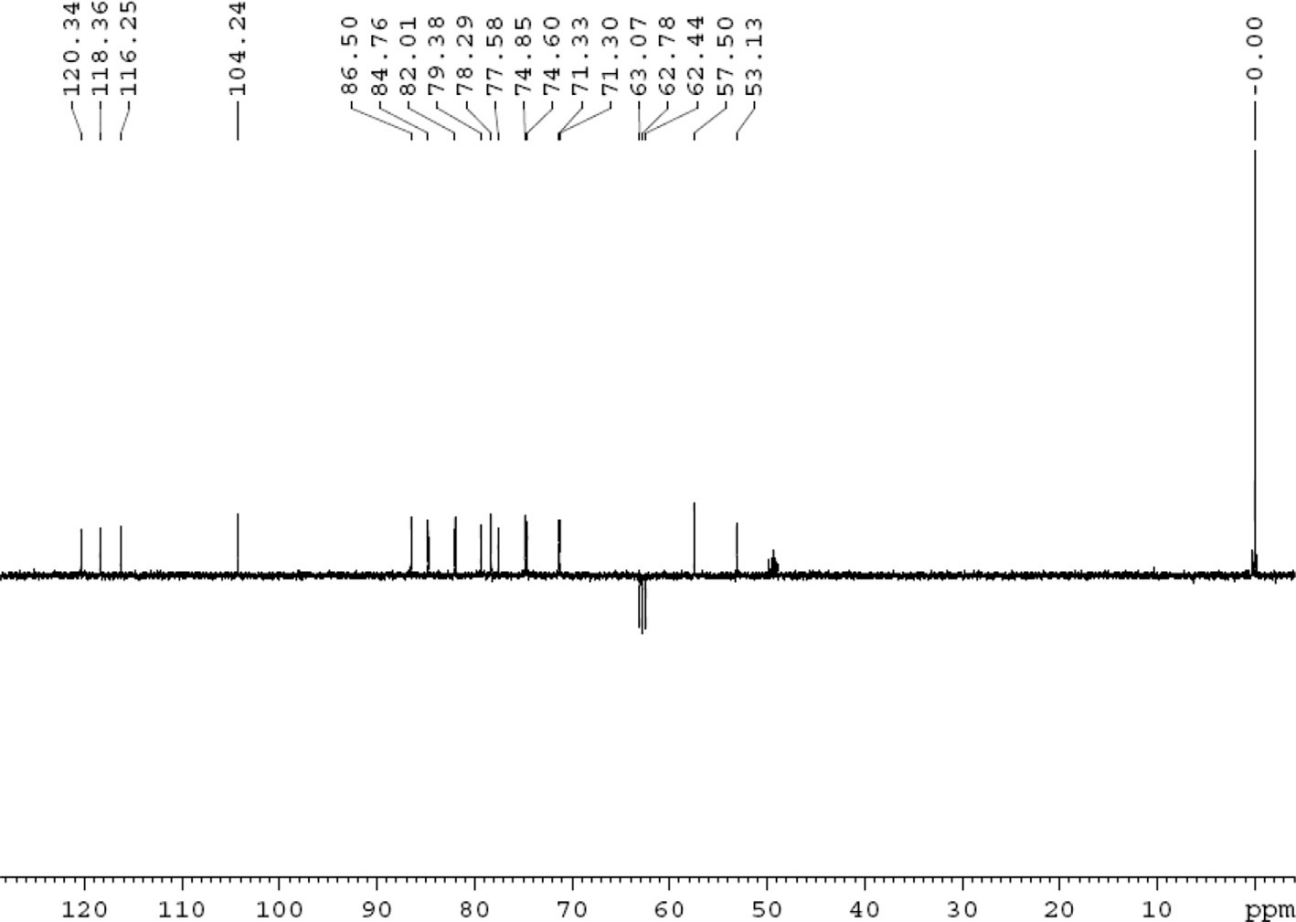
DEPT 135 (CD_3_OD) spectrum of compound **5**.

**Fig. 32:**
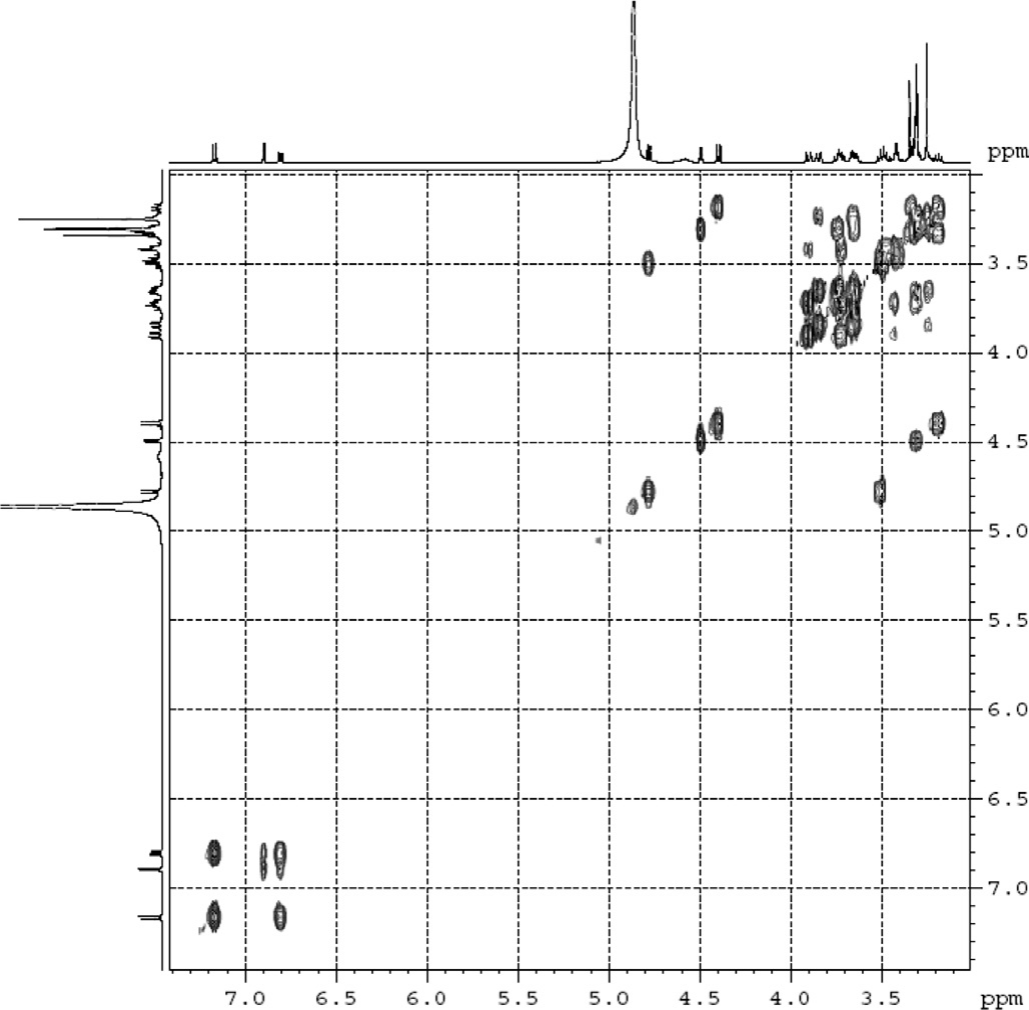
^1^H ^1^H COSY (CD_3_OD) spectrum of compound **5**.

**Fig. 33:**
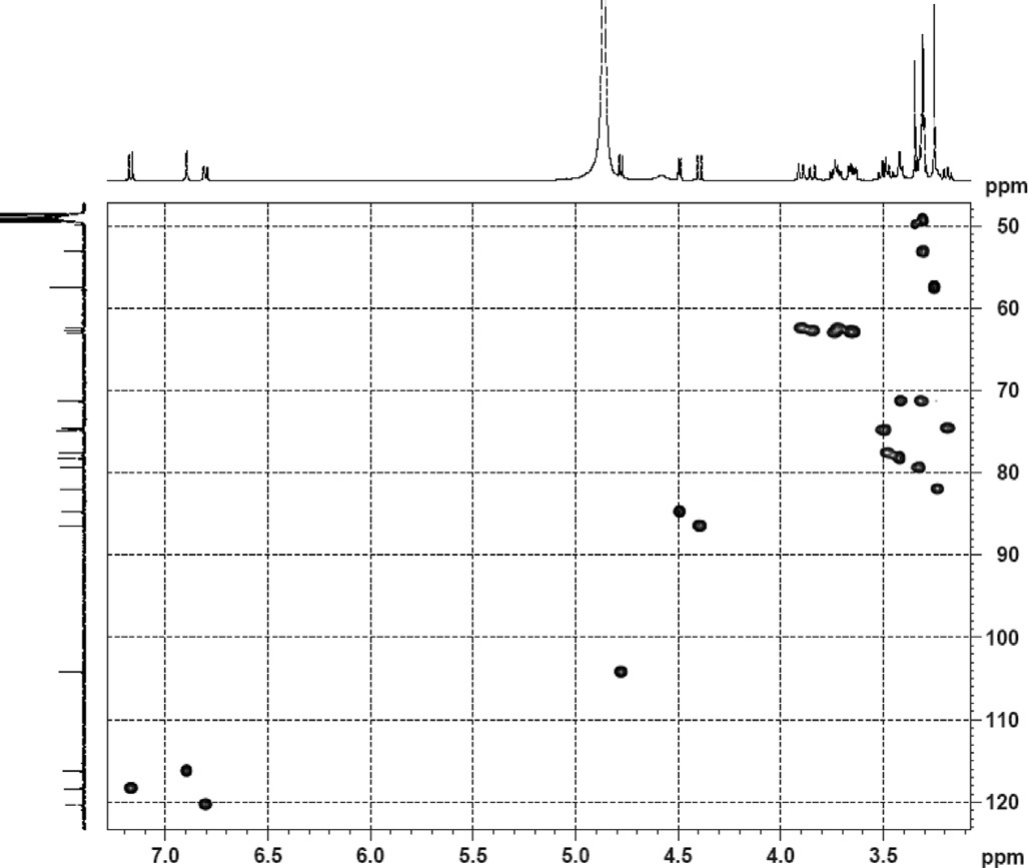
HSQC (CD_3_OD) spectrum of compound **5**.

**Fig. 34:**
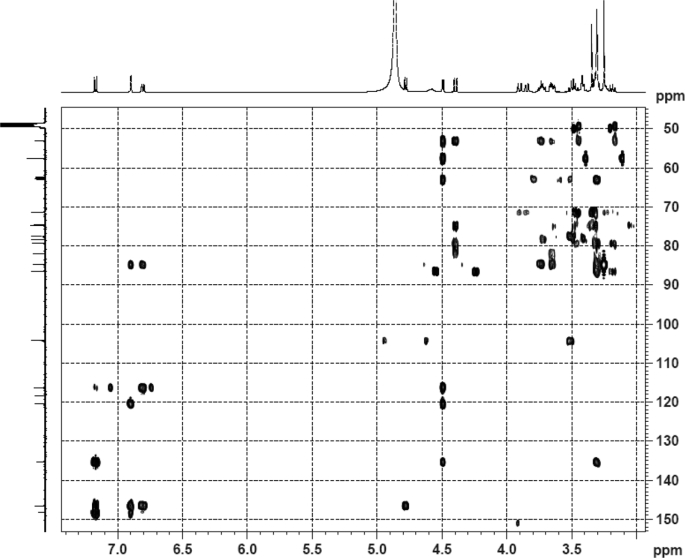
HMBC (CD_3_OD) spectrum of compound **5**.

**Fig. 35:**
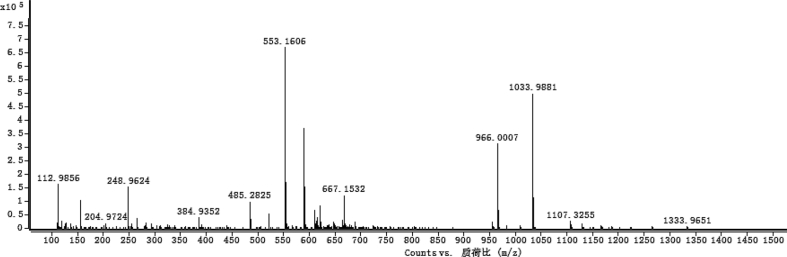
HRESI-TOF-MS spectrum of compound **5**.

**Fig. 36:**
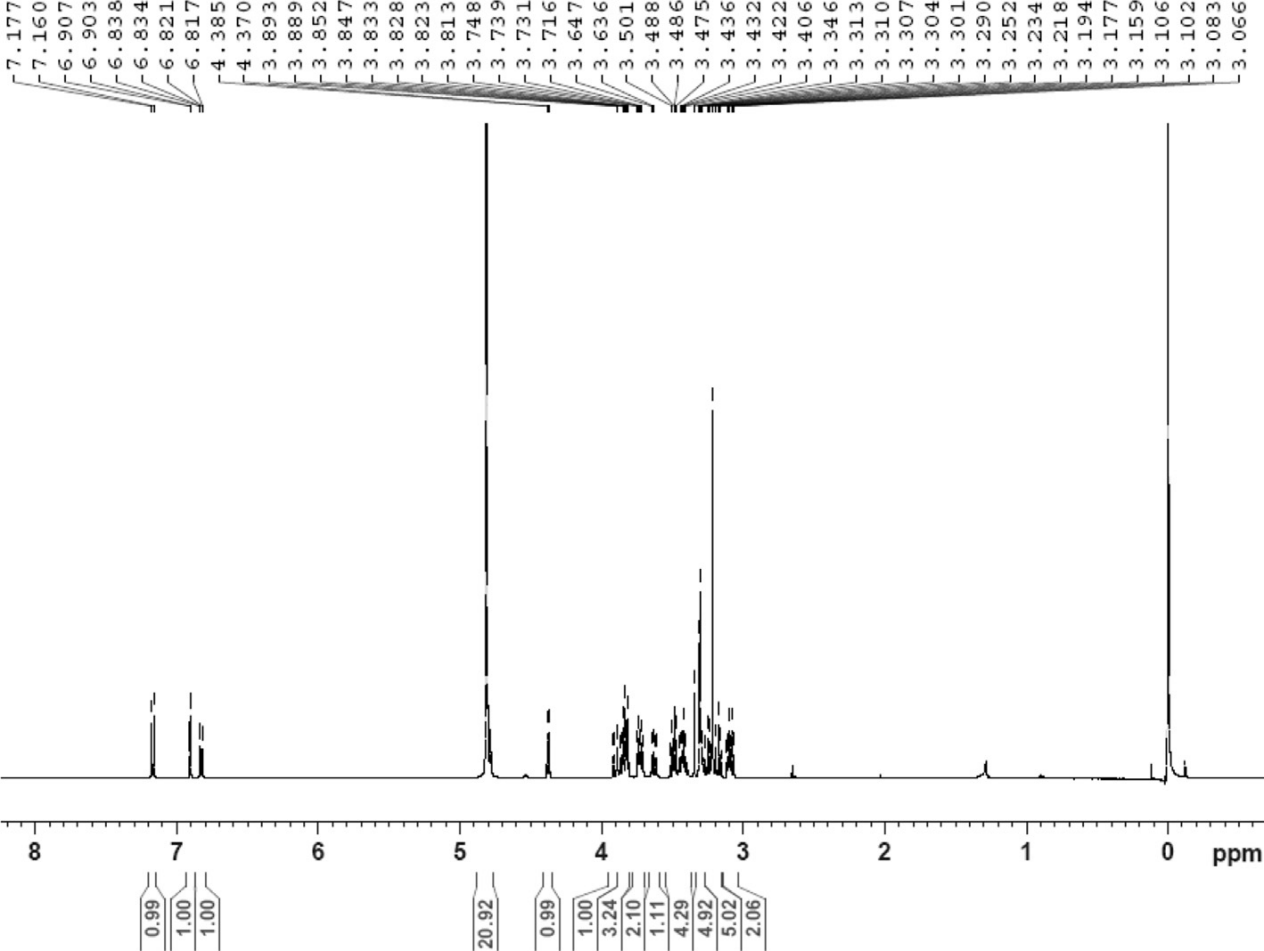
^1^H NMR (500 MHz, CD_3_OD) spectrum of compound **6**.

**Fig. 37:**
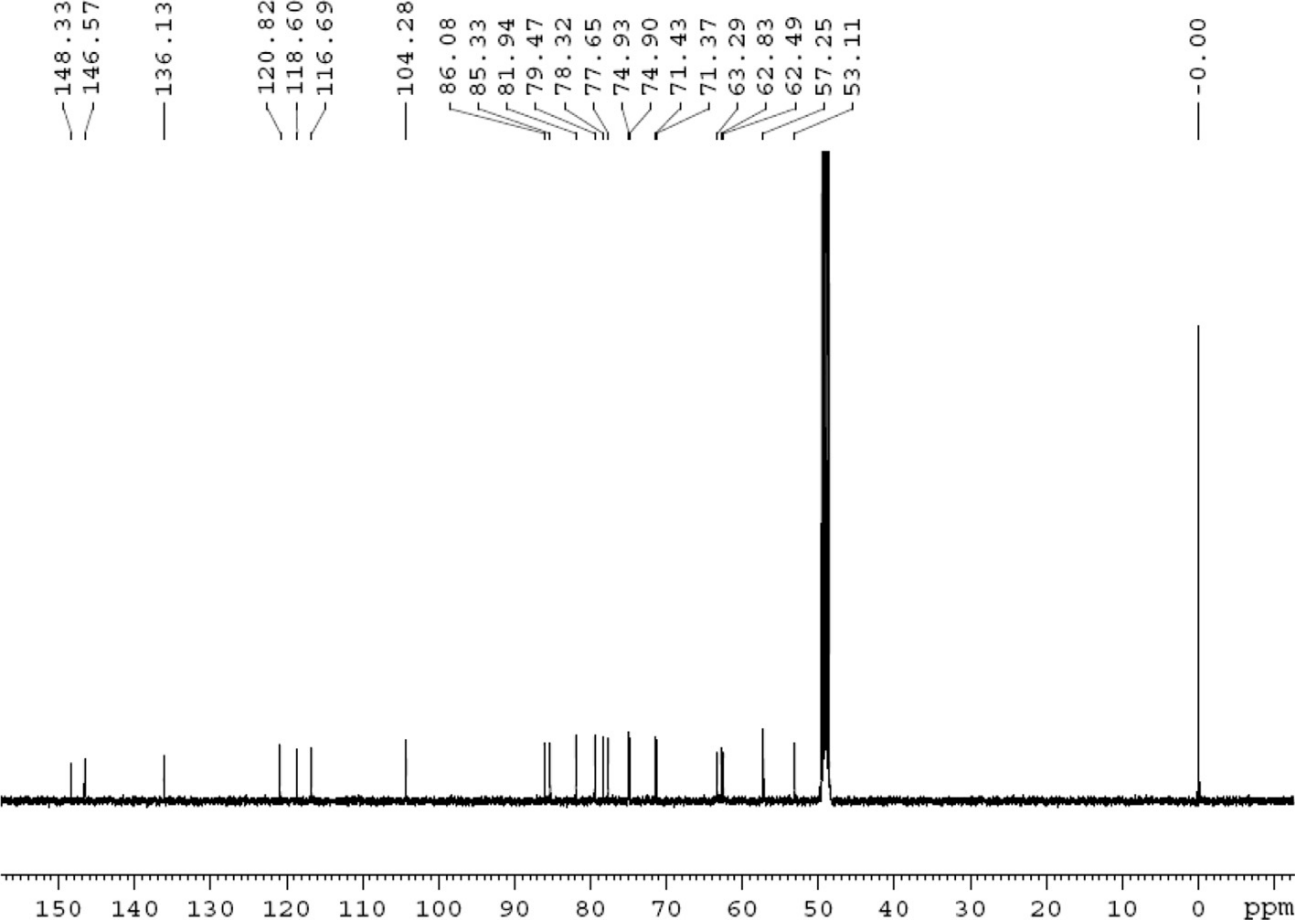
^13^C NMR (125 MHz, CD_3_OD) spectrum of compound **6**.

**Fig. 38:**
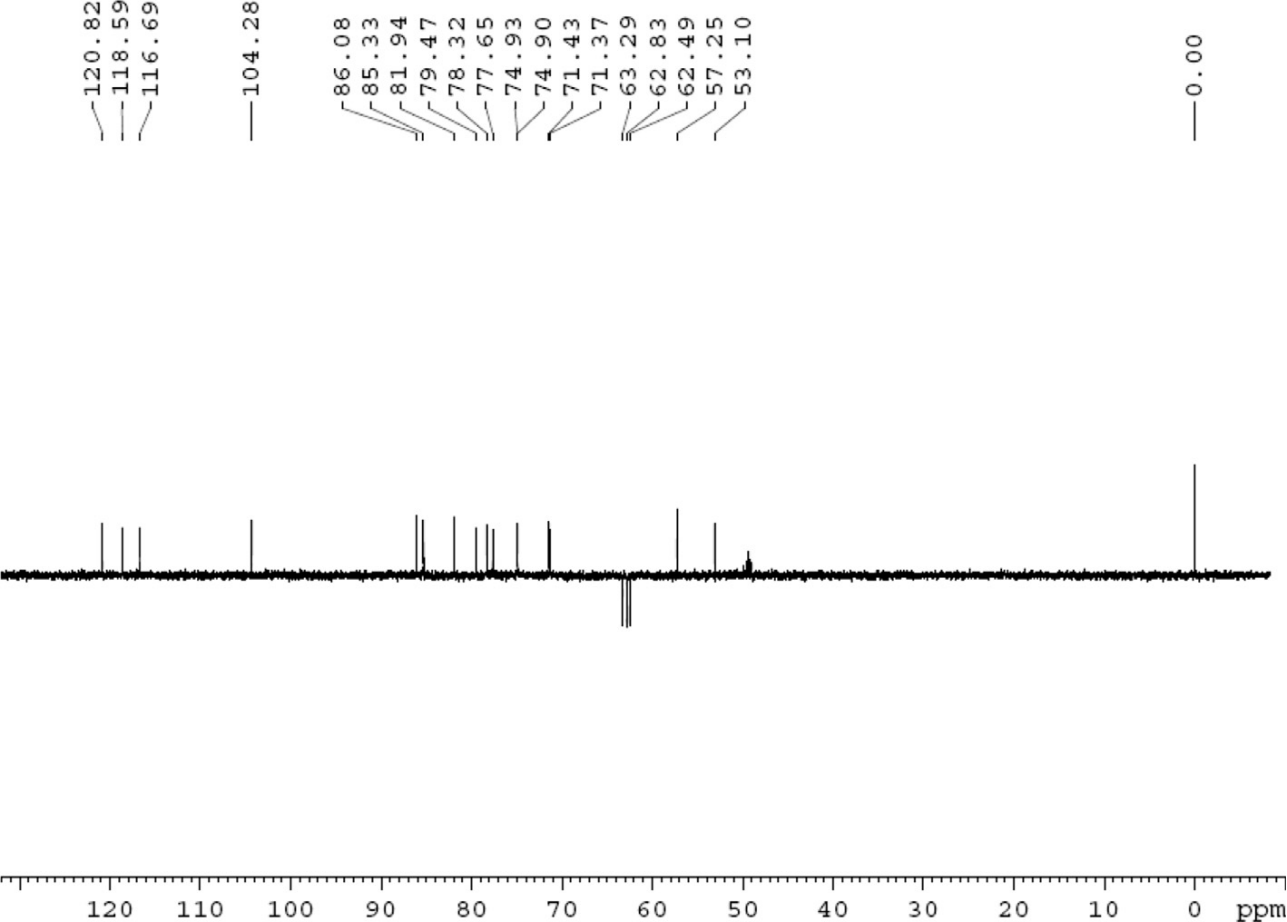
DEPT 135 (CD_3_OD) spectrum of compound **6**.

**Fig. 39:**
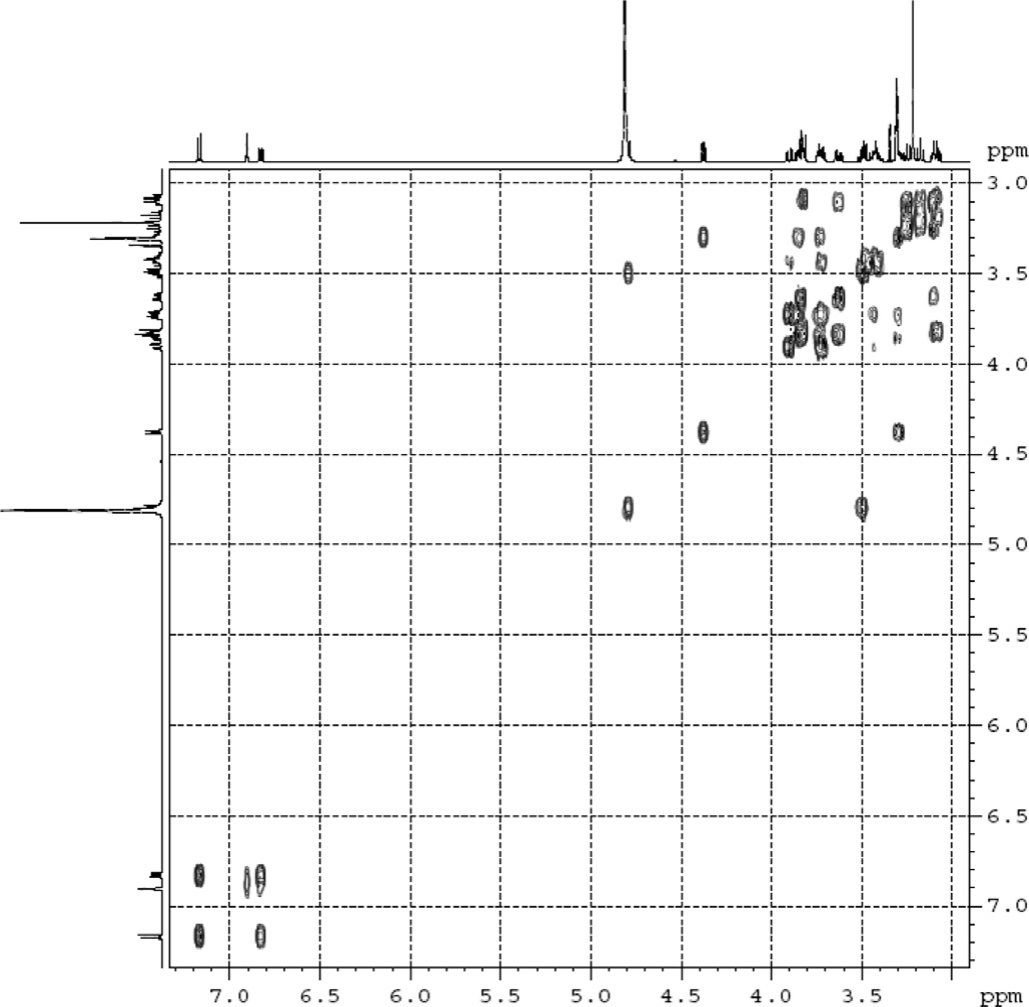
^1^H ^1^H COSY (CD_3_OD) spectrum of compound **6**.

**Fig. 40:**
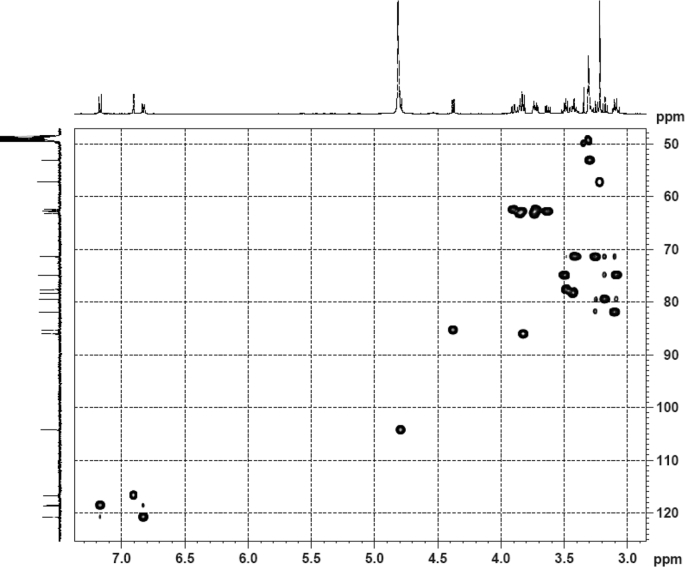
HSQC (CD_3_OD) spectrum of compound **6**.

**Fig. 41:**
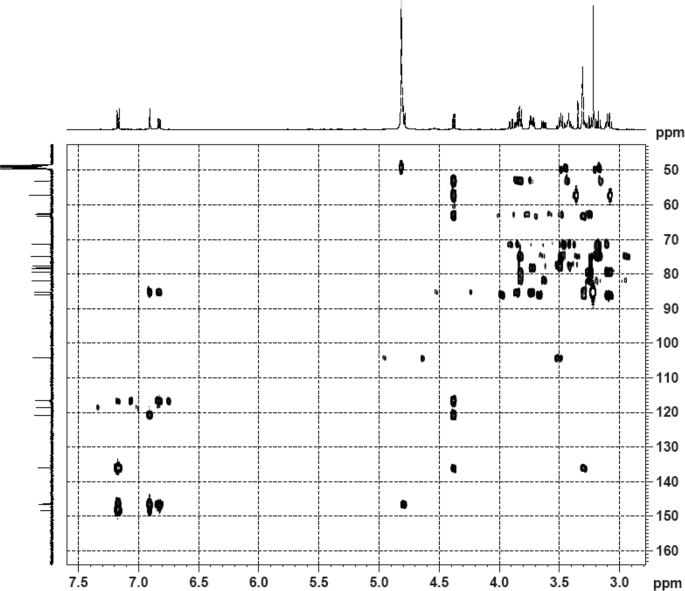
HMBC (CD_3_OD) spectrum of compound **6**.

**Fig. 42:**
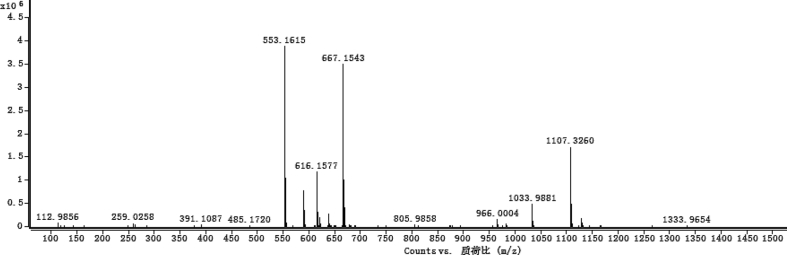
HRESI-TOF-MS spectrum of compound **6**.

**Fig. 43:**
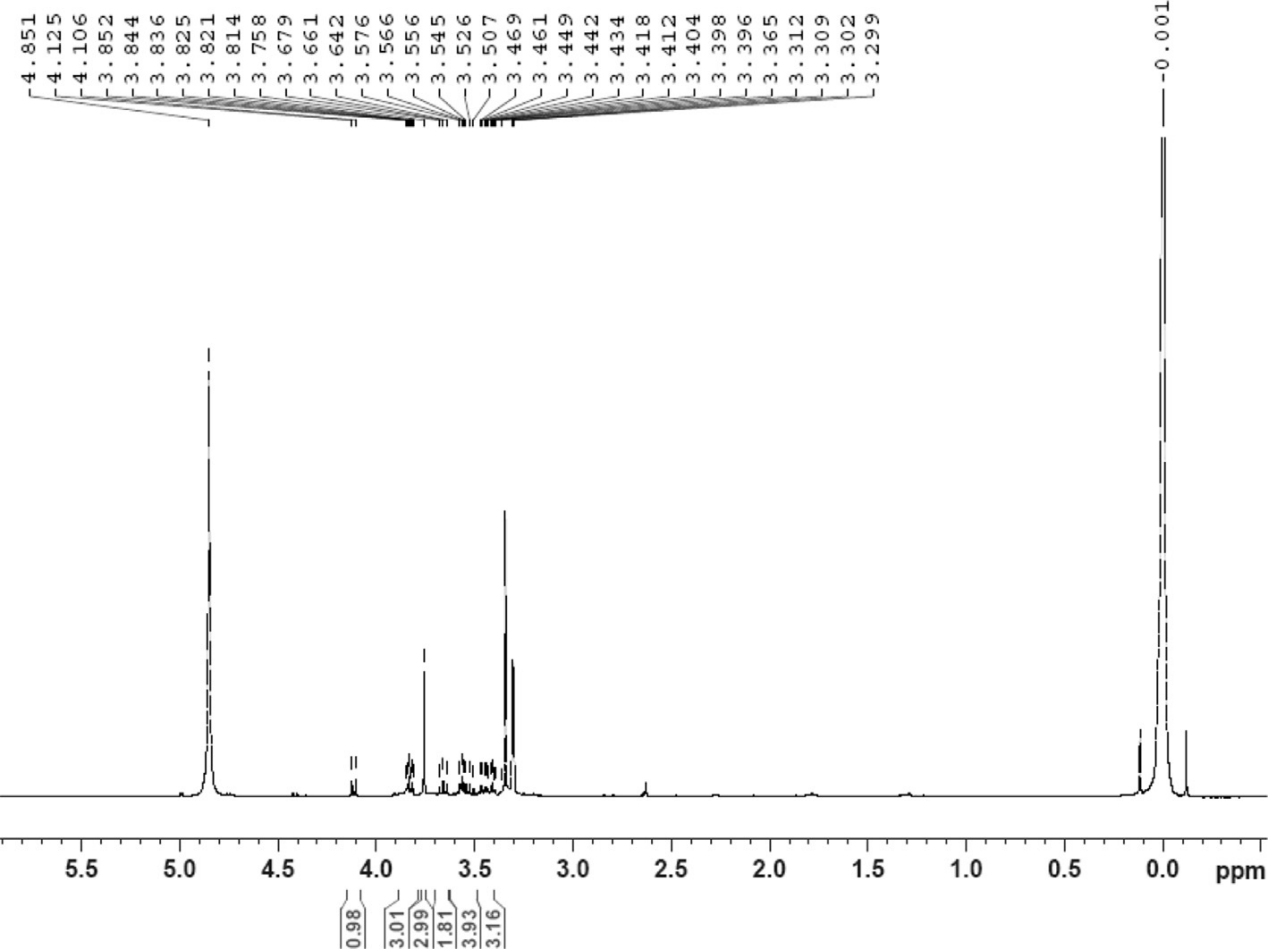
^1^H NMR (500 MHz, CD_3_OD) spectrum of compound **7**.

**Fig. 44:**
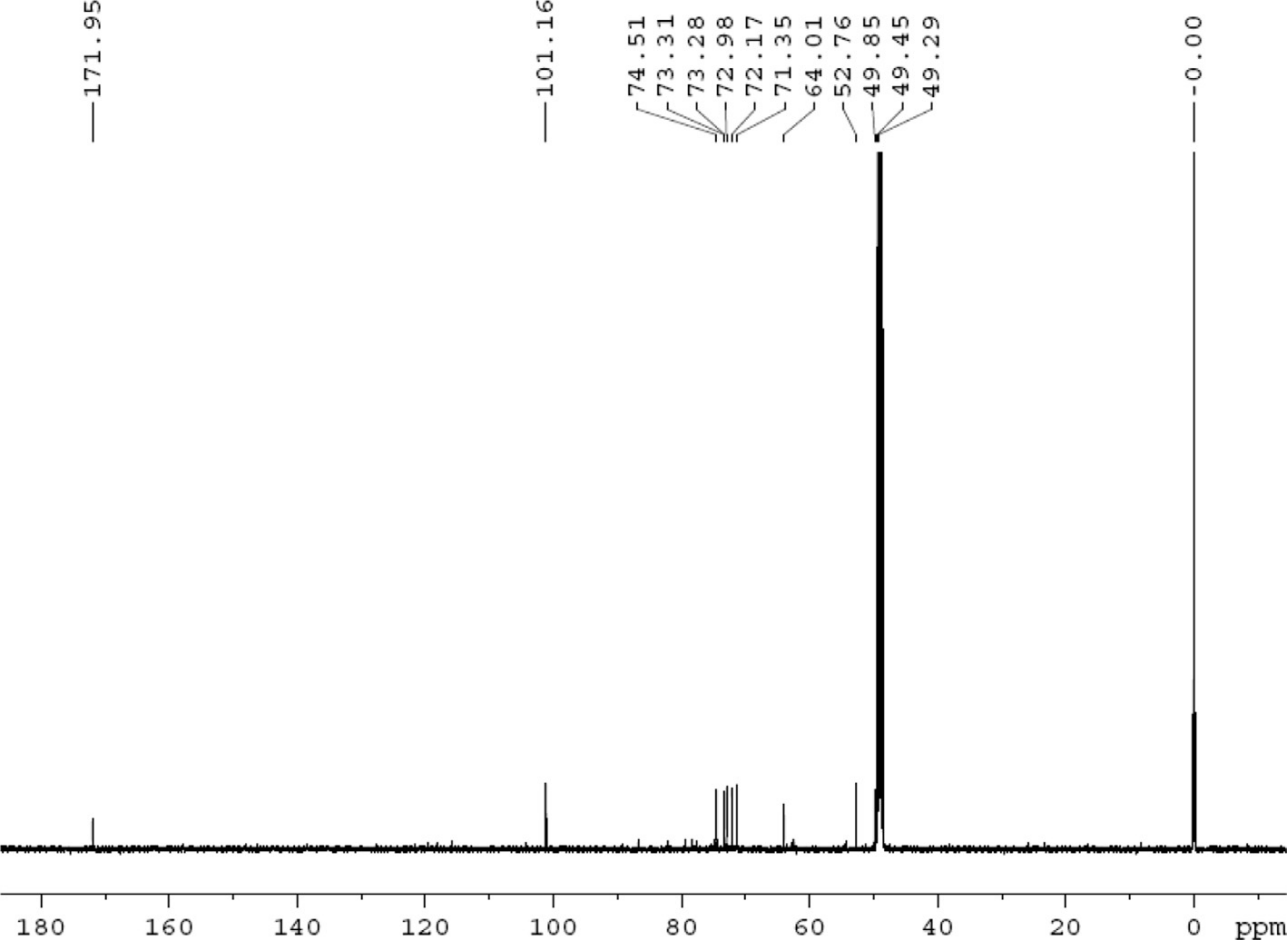
^13^C NMR (125 MHz, CD_3_OD) spectrum of compound **7**.

**Fig. 45:**
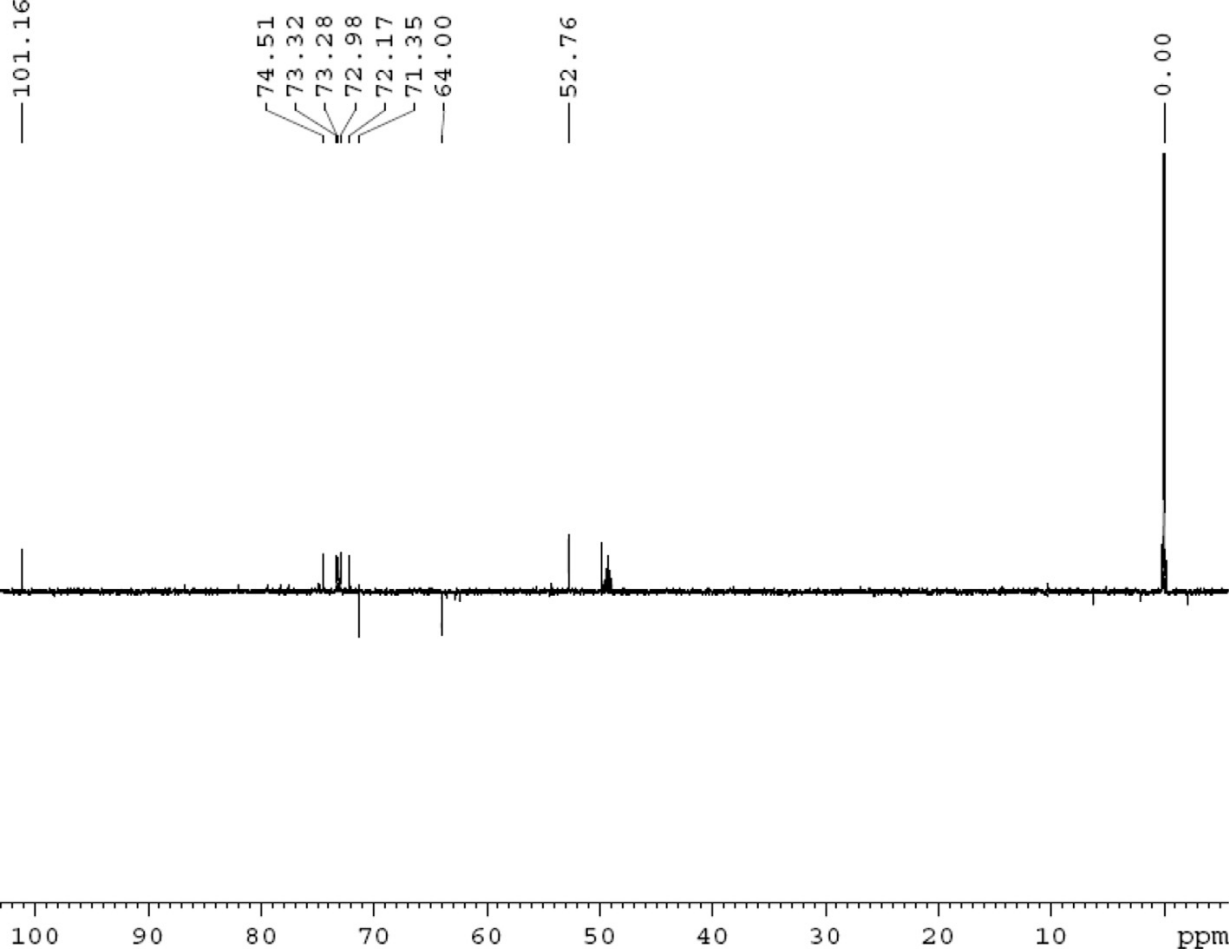
DEPT 135 (CD_3_OD) spectrum of compound **7**.

**Fig. 46:**
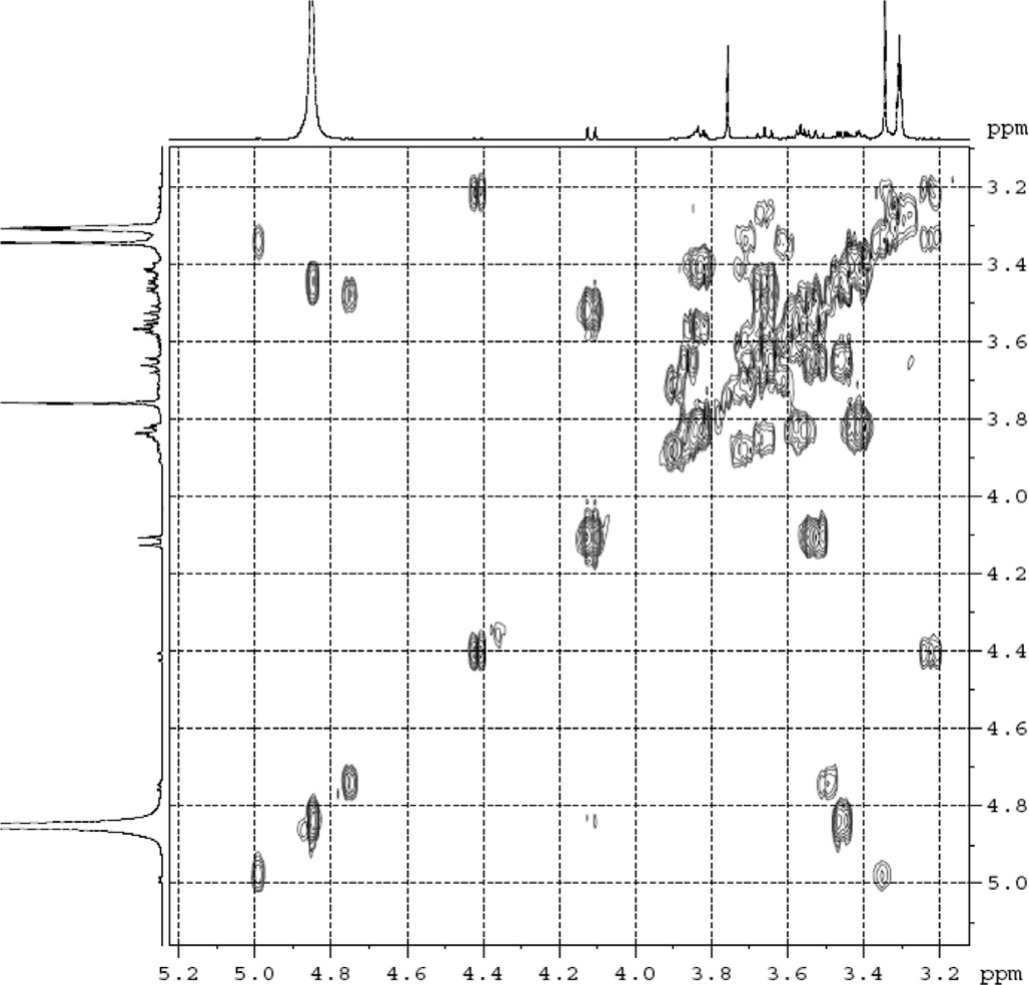
^1^H ^1^H COSY (CD_3_OD) spectrum of compound **7**.

**Fig. 47:**
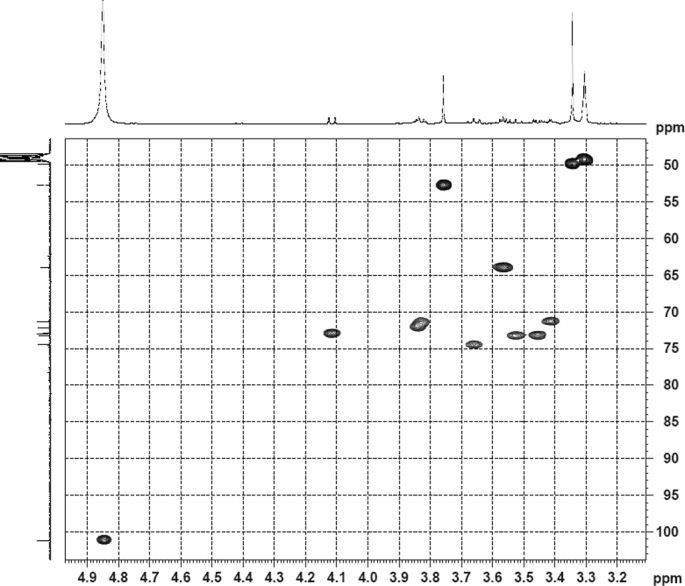
HSQC (CD_3_OD) spectrum of compound **7**.

**Fig. 48:**
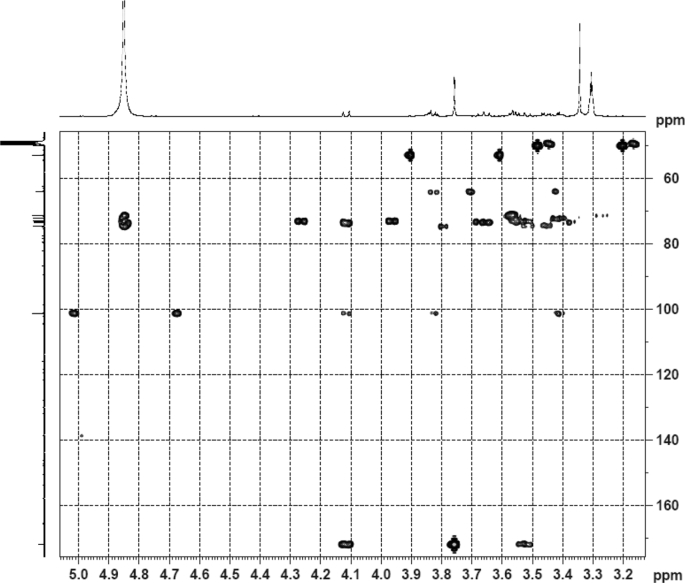
HMBC (CD_3_OD) spectrum of compound **7**.

**Fig. 49:**
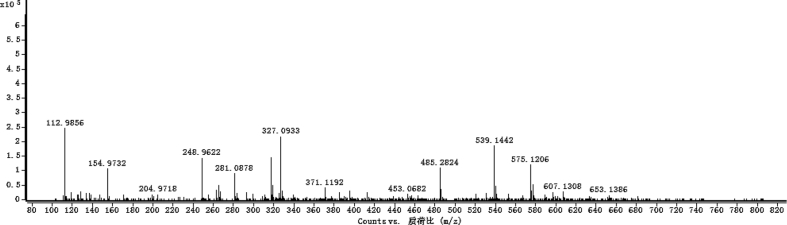
HRESI-TOF-MS spectrum of compound **7**.

**Fig. 50:**
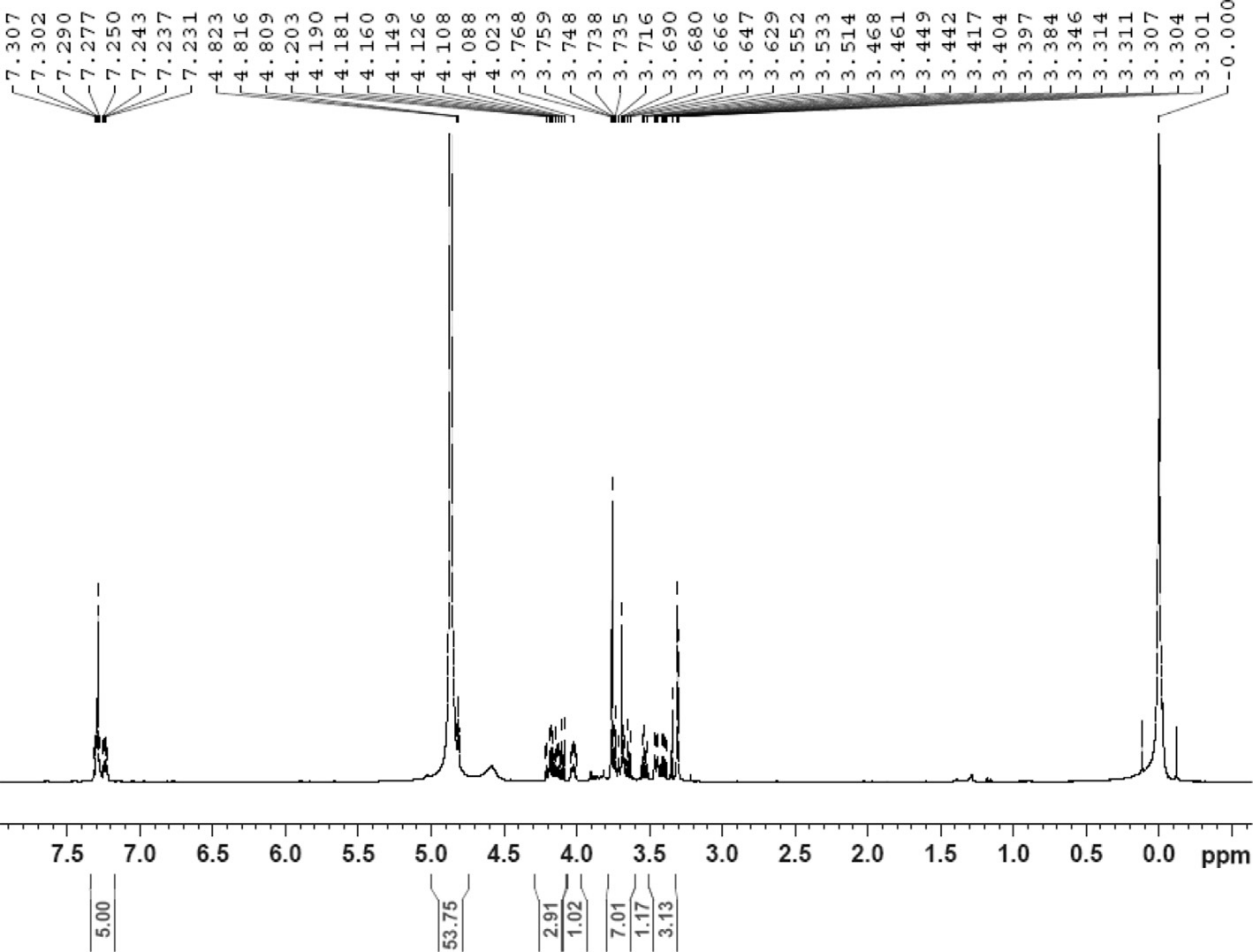
^1^H NMR (500 MHz, CD_3_OD) spectrum of compound **8**.

**Fig. 51:**
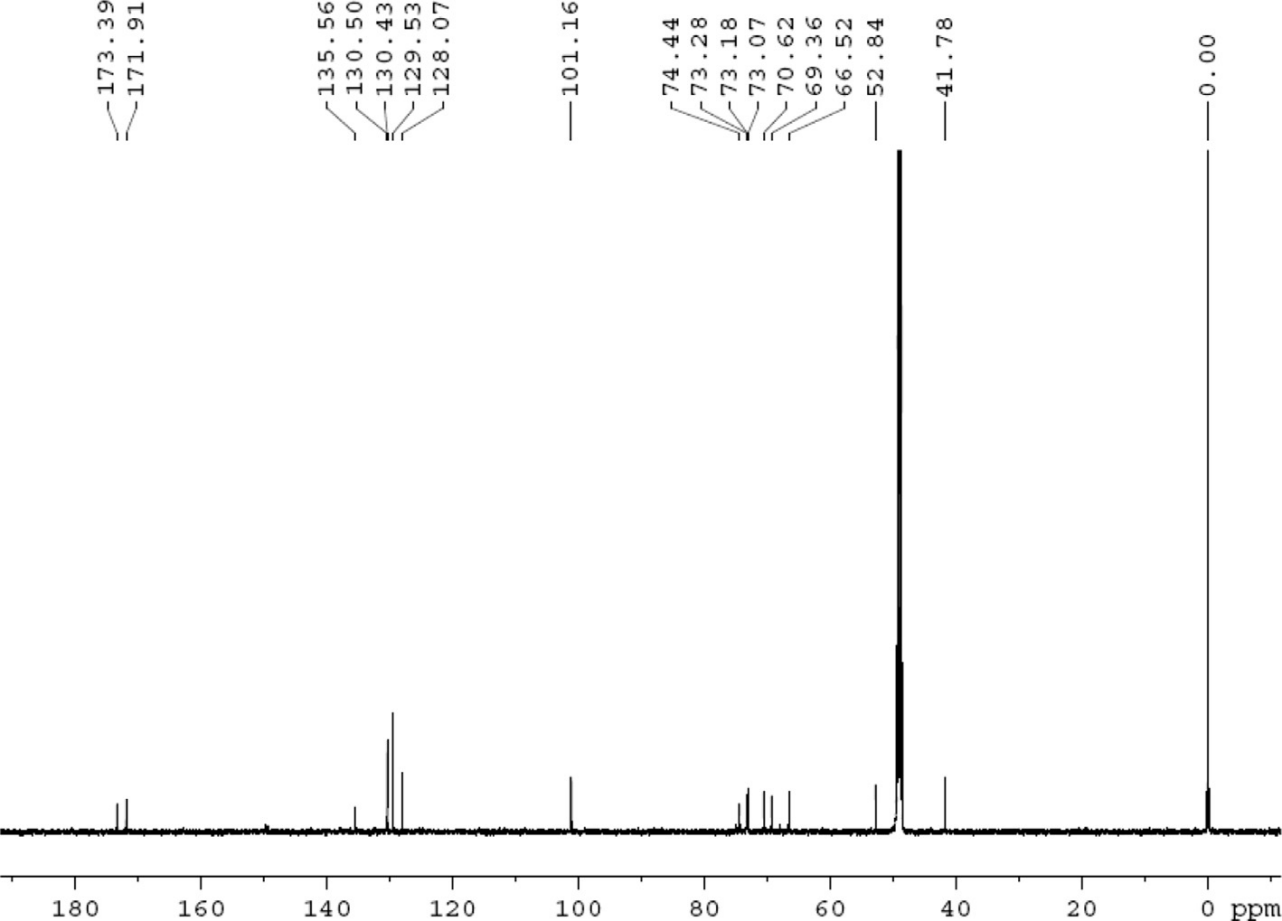
^13^C NMR (125 MHz, CD_3_OD) spectrum of compound **8**.

**Fig. 52:**
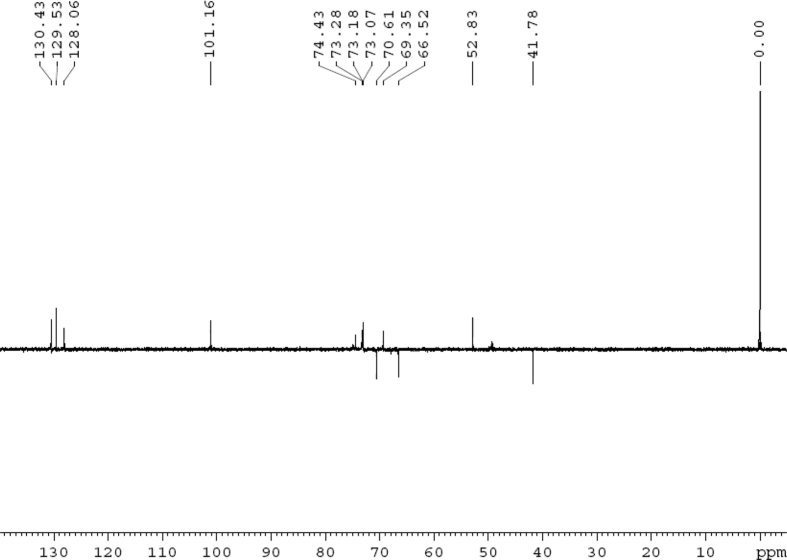
DEPT 135 (CD_3_OD) spectrum of compound **8**.

**Fig. 53:**
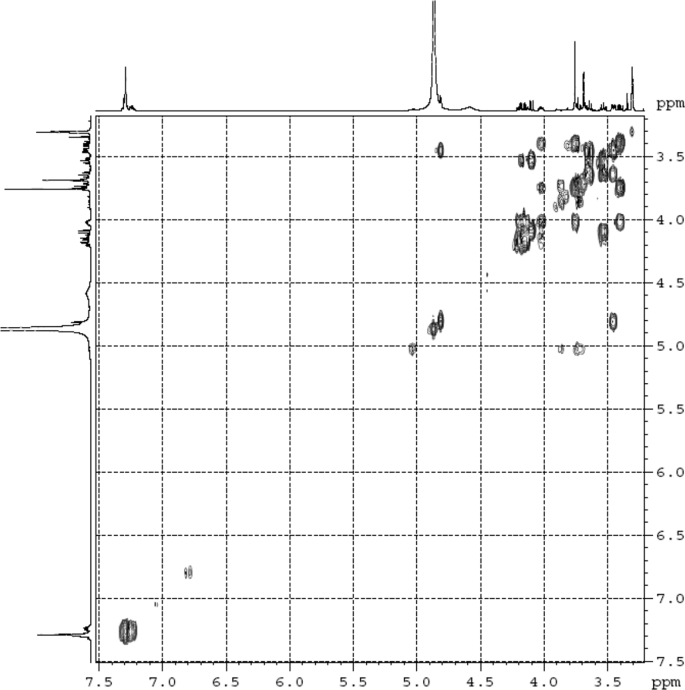
^1^H ^1^H COSY (CD_3_OD) spectrum of compound **8**.

**Fig. 54:**
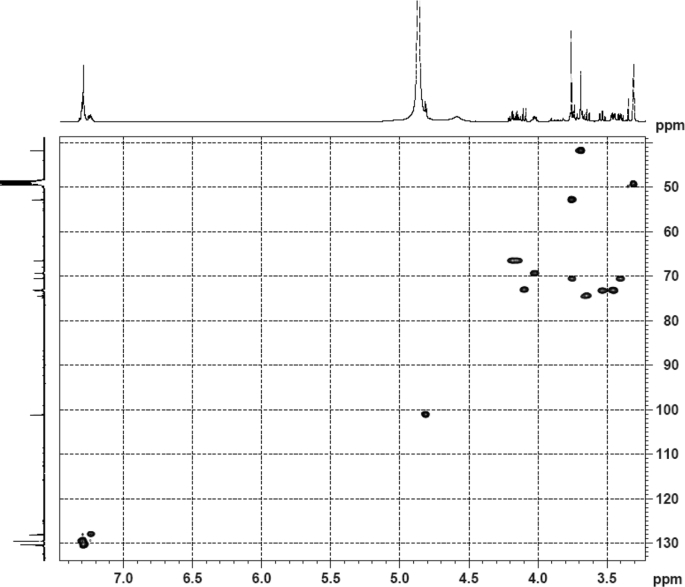
HSQC (CD_3_OD) spectrum of compound **8**.

**Fig. 55:**
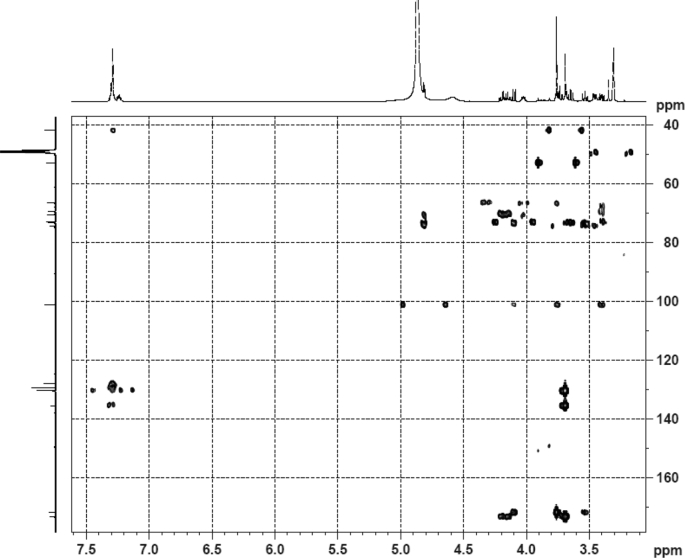
HMBC (CD_3_OD) spectrum of compound **8**.

**Fig. 56:**
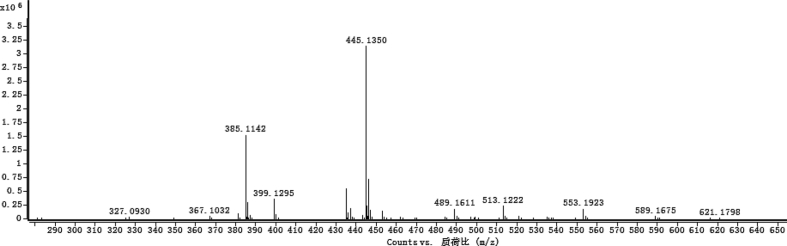
HRESI-TOF-MS spectrum of compound **8**.

## 2 Experimental design, materials, and methods

### 2.1 Study area description

*Pugionium cornutum* L. Gaertn (PC), Cruciferae family, is widely distributed in the Badain Jaran Desert, Kubuqi Desert, Mu Us Desert, Horqin sandy land, and Hulunbuir sandy land [2]. As the traditional arenicolous Mongolian medicine, PC has accumulated rich experience and knowledge of promoting gastrointestinal motility and improving indigestion [3]. Previously, a further phytochemical investigation on the 70% ethanol-water extract of the roots of *P. cornutum* led to the isolation and characterization of eight compounds that have been not reported previously. Among them, **1**–**4** were rare naturally occurring thiohydantoin derivatives. Herein, their structure characterization has been identified by various spectrometry methods including NMR and MS spectra.

### 2.2 Plant material

The roots of *Pugionium cornutum* L. Gaertn were collected from Alxa Youqi, Inner Mongolia Autonomous region, China, and identified by Dr. Li Tianxiang (Experiment Teaching Department, Tianjin University of Traditional Chinese Medicine). The voucher specimen was deposited at the Academy of Traditional Chinese Medicine of Tianjin University of TCM.

### 2.3 NMR and MS spectrum of the isolates

The dried roots of PC were refluxed with 70% ethanol-water for three times. Evaporation of the solvent under pressure provided a 70% ethanol-water extract, which was partitioned with H_2_O and EtOAc to gain H_2_O layer and EtOAc layer partition, respectively. The H_2_O layer partition was subjected to D101 macroporous resin CC and eluted with H_2_O, 95% EtOH, and acetone, successively. Then 95% EtOH eluate and EtOAc layer partition was isolated by silica gel, Sephadex LH-20 chromatography columns and preparative HPLC. As results, four new rare thiohydantoin derivatives pugcornols A (**1**), B (**2**), C (**3**), and D (**4**) [[4], [5], [6], [7]], two new glucosinolates pugcornosides A (**5**) [[8], [9], [10]], B (**6**) [10], two new others pugcornosides C (**7**) [11], D (**8**) were obtained and identified by using NMR, MS, ECD technologies, as well as chemical reaction.

#### 2.3.1 Pugcornol A (**1**)

Colorless oil; [*α*]_D_^25^ –31.3 (*conc.* 0.12, MeOH); CD (*conc*. 0.0015 M, MeOH) mdeg (*λ*_nm_): –1.39 (235), −3.33 (256), −3.60 (269); CD (*conc*. 0.0015 M, CH_3_CN) mdeg (*λ*_nm_): –1.21 (229), −2.83 (245), −4.52 (267); UV *λ*_max_ (MeOH) nm (log *ε*): 269 (3.81); IR *ν*_max_ (KBr) cm^−1^: 3364, 2943, 1750, 1424, 1347, 1225, 1124, 1061, 1025; ^1^H NMR (DMSO‑*d*_6_, 500 MHz) and ^13^C NMR (DMSO‑*d*_6_, 125 MHz) data see Fig. 1, Fig. 2, Fig. 3, Fig. 4, Fig. 5, Fig. 6. HRESI-TOF-MS data see Fig. 7.

#### 2.3.2 Pugcornol B (**2**)

Colorless oil; [*α*]_D_^25^ –39.3 (*conc.* 0.12, MeOH); CD (*conc*. 0.0015 M, MeOH) mdeg (*λ*_nm_): –1.30 (223), −5.34 (270); CD (*conc*. 0.0015 M, CH_3_CN) mdeg (*λ*_nm_): –1.56 (223), −6.80 (252), −4.81 (272); UV *λ*_max_ (MeOH) nm (log *ε*): 277 (3.96); IR *ν*_max_ (KBr) cm^−1^: 3355, 2941, 1750, 1428, 1346, 1236, 1133, 1049, 1010; ^1^H NMR (CD_3_OD, 500 MHz) and ^13^C NMR (CD_3_OD, 125 MHz) data Fig. 8, Fig. 9, Fig. 10, Fig. 11, Fig. 12, Fig. 13. HRESI-TOF-MS data see Fig. 14.

#### 2.3.3 Pugcornol C (**3**)

Colorless oil; [*α*]_D_^25^ –36.1 (*conc.* 0.14, MeOH); CD (*conc*. 0.0015 M, MeOH) mdeg (*λ*_nm_): –0.34 (228), −2.60 (253), −2.99 (269); CD (*conc*. 0.0015 M, CH_3_CN) mdeg (*λ*_nm_): –1.26 (231), −3.91 (250), −5.16 (267); UV *λ*_max_ (MeOH) nm (log *ε*): 249 (3.50), 268 (3.58); IR *ν*_max_ (KBr) cm^−1^: 3365, 2935, 1749, 1419, 1347, 1236, 1133, 1050, 1015; ^1^H NMR (CD_3_OD, 500 MHz) and ^13^C NMR (CD_3_OD, 125 MHz) data Fig. 14, Fig. 15, Fig. 16, Fig. 17, Fig. 18, Fig. 19, Fig. 20. HRESI-TOF-MS data see Fig. 21.

#### 2.3.4 Pugcornol D (**4**)

Colorless oil; [*α*]_D_^25^ –64.1 (*conc*. 0.13, MeOH); CD (*conc*. 0.0015 M, MeOH) mdeg (*λ*_nm_): –35.54 (214), +14.06 (238), +0.12 (263); CD (*conc*. 0.0015 M, CH_3_CN) mdeg (*λ*_nm_): –33.13 (212), 12.47 (238), +0.23 (262); UV *λ*_max_ (MeOH) nm (log *ε*): 214 (3.44), 267 (2.38); IR *ν*_max_ (KBr) cm^−1^: 3420, 2943, 1765, 1701, 1447, 1419, 1361, 1014; ^1^H NMR (CD_3_OD, 500 MHz) and ^13^C NMR (CD_3_OD, 125 MHz) data Fig. 22, Fig. 23, Fig. 24, Fig. 25, Fig. 26, Fig. 27. HRESI-TOF-MS data see Fig. 28.

#### 2.3.5 Pugcornoside A (**5**)

White powder; [*α*]_D_^25^ –23.2 (*conc.* 0.11, MeOH); UV *λ*_max_ (MeOH) nm (log *ε*): 223 (3.61), 277 (3.18); IR *ν*_max_ (KBr) cm^−1^: 3365, 2922, 1646, 1558, 1507, 1457, 1277, 1070; ^1^H NMR (CD_3_OD, 500 MHz) and ^13^C NMR (CD_3_OD, 125 MHz) data Fig. 29, Fig. 30, Fig. 31, Fig. 32, Fig. 33, Fig. 34. HRESI-TOF-MS data see Fig. 35.

#### 2.3.6 Pugcornoside B (**6**)

White powder; [*α*]_D_^25^ –52.6 (*conc.* 0.13, MeOH); UV *λ*_max_ (MeOH) nm (log *ε*): 220 (3.65), 277 (3.18); IR *ν*_max_ (KBr) cm^−1^: 3366, 2918, 1600, 1507, 1456, 1436, 1278, 1045, 1072; ^1^H NMR (CD_3_OD, 500 MHz) and ^13^C NMR (CD_3_OD, 125 MHz) data Fig. 36, Fig. 37, Fig. 38, Fig. 39, Fig. 40, Fig. 41. HRESI-TOF-MS data see Fig. 42.

#### 2.3.7 Pugcornoside C (**7**)

White powder; [*α*]_D_^25^ +6.3 (*conc*. 0.12,MeOH); UV *λ*_max_ (MeOH) nm (log *ε*): 266 (2.94); IR *ν*_max_ (KBr) cm^−1^: 3386, 2391, 1734, 1652, 1107, 1044; ^1^H NMR (CD_3_OD, 500 MHz) and ^13^C NMR (CD_3_OD, 125 MHz) data Fig. 43, Fig. 44, Fig. 45, Fig. 46, Fig. 47, Fig. 48. HRESI-TOF-MS data see Fig. 49.

#### 2.3.8 Pugcornoside D (**8**)

White powder; [*α*]_D_^25^ +28.1 (*conc*. 0.12, MeOH); UV *λ*_max_ (MeOH) nm (log *ε*): 205 (3.72), 262 (2.77); IR *ν*_max_ (KBr) cm^−1^: 3420, 2933, 1733, 1635, 1466, 1339, 1267, 1154, 1112, 1047; ^1^H NMR (CD_3_OD, 500 MHz) and ^13^C NMR (CD_3_OD, 125 MHz) data Fig. 50, Fig. 51, Fig. 52, Fig. 53, Fig. 54, Fig. 55. HRESI-TOF-MS data see Fig. 56.
